# Clinicopathologic and gene expression parameters predict liver cancer prognosis

**DOI:** 10.1186/1471-2407-11-481

**Published:** 2011-11-09

**Authors:** Ke Hao, John Lamb, Chunsheng Zhang, Tao Xie, Kai Wang, Bin Zhang, Eugene Chudin, Nikki P Lee, Mao Mao, Hua Zhong, Danielle Greenawalt, Mark D Ferguson, Irene O Ng, Pak C Sham, Ronnie T Poon, Cliona Molony, Eric E Schadt, Hongyue Dai, John M Luk

**Affiliations:** 1 Merck Research Laboratories, Boston, MA, USA; 2 Department of Surgery, University of Hong Kong, Pokfulam, Hong Kong SAR, China; 3 Department of Pathology, The University of Hong Kong, Pokfulam, Hong Kong SAR, China; 4 Department of Psychiatry and Genome Research Center, The University of Hong Kong, Pokfulam, Hong Kong SAR, China; 5 Departments of Pharmacology and Surgery and Cancer Science Institute, National University of Singapore, Singapore

## Abstract

**Background:**

The prognosis of hepatocellular carcinoma (HCC) varies following surgical resection and the large variation remains largely unexplained. Studies have revealed the ability of clinicopathologic parameters and gene expression to predict HCC prognosis. However, there has been little systematic effort to compare the performance of these two types of predictors or combine them in a comprehensive model.

**Methods:**

Tumor and adjacent non-tumor liver tissues were collected from 272 ethnic Chinese HCC patients who received curative surgery. We combined clinicopathologic parameters and gene expression data (from both tissue types) in predicting HCC prognosis. Cross-validation and independent studies were employed to assess prediction.

**Results:**

HCC prognosis was significantly associated with six clinicopathologic parameters, which can partition the patients into good- and poor-prognosis groups. Within each group, gene expression data further divide patients into distinct prognostic subgroups. Our predictive genes significantly overlap with previously published gene sets predictive of prognosis. Moreover, the predictive genes were enriched for genes that underwent normal-to-tumor gene network transformation. Previously documented liver eSNPs underlying the HCC predictive gene signatures were enriched for SNPs that associated with HCC prognosis, providing support that these genes are involved in key processes of tumorigenesis.

**Conclusion:**

When applied individually, clinicopathologic parameters and gene expression offered similar predictive power for HCC prognosis. In contrast, a combination of the two types of data dramatically improved the power to predict HCC prognosis. Our results also provided a framework for understanding the impact of gene expression on the processes of tumorigenesis and clinical outcome.

## Background

Hepatocellular carcinoma (HCC) is the fifth most common cancer in the world, accounting for approximately one million deaths, with an increasing trend of new incidences annually [1-3]. Surgical resection is regarded as the standard curative treatment of HCC [3]. However, prognosis following surgery varies substantially. This variation becomes a hurdle in searching for effective and efficacious therapies and cancer management strategies. There is an ongoing search for predictive biomarkers of cancer prognosis, where pathological parameters, protein biomarkers, mRNA expression levels, and genomic DNA abnormalities have been surveyed [4-9].

On two independent Hong Kong HCC cohorts that we previously described [10], the HCC prognosis was significantly associated with clinicopathologic parameters including tumor size, number of tumor nodules (NOTN), tumor stage (new AJCC and pTNM), venous infiltration status, serum albumin level (ALBU), and serum α-fetoprotein level (AFP). These parameters were further summarized into a linear score that was demonstrated to partially predict disease-free survival ([DFS] time to tumor recurrence) and overall survival (time to death) [10]. A natural path to further enhance this prediction model would be to incorporate molecular level biomarkers, for example, gene expression profiles in the tumor or adjacent normal tissues. Currently, such efforts have been limited due to the availability of fresh frozen tissues [4] forcing some studies to use paraffin-embedded samples [9,11]. Most importantly, the search for gene signatures should be conducted by conditioning on the clinicopathologic parameters, and focus on the identification of novel variance components that improve the prognosis prediction beyond that achieved by the clinicopathologic features alone.

Herein we carried out a carefully designed search for gene expression signatures underlying prognosis of HCC using tumor and adjacent normal tissue expression profiles. We identify a gene expression signature that significantly enhances our ability to predict HCC prognosis. Additionally, we demonstrate that this HCC prognosis signature is related to widespread changes we previously identified in the liver tissue network that are associated with HCC, providing additional mechanistic insights into tumorigenesis.

## Methods

### Study Subjects

A total of 272 HCC patients were included in the initial training dataset. Resected tumor and adjacent non-tumor liver tissues were collected from patients who had undergone hepatectomy for curative treatment of HCC at Queen Mary Hospital, Pokfulam, Hong Kong between 1993 and 2007 [10]. Informed consents were obtained from patients regarding to the use of the liver specimens for research. Additional File 1, Table S1 summarized the demographic and clinicopathologic features of these patients. All samples came from individuals who provided written informed consent to make their samples available for scientific research. In addition, all of the samples and patient data were approved for use in this study by IRBs specific to each of the participating organizations. Experimental research reported in this paper was also approved by IRBs of each participating organizations. Research reported in this paper was in compliance with the Helsinki Declaration.

### Pathology Parameter Measurements

The clinicopathologic features of the patients analyzed were sex, age, tumor size, number of tumor nodules, cellular differentiation according to the Edmondson classification, venous infiltration without differentiation into portal or hepatic venules, tumor node metastasis stage (pTNM and AJCC), serum hepatitis B surface antigen (HBsAg) status, and background liver disease in non-tumorous liver tissue [12].

### Sample processing to isolate RNA

Fresh frozen tissue was placed in a chilled milling tube along with a stainless steel bead, dipped in a liquid nitrogen bath and loaded onto the QIAGEN TissueLyser for milling (30 Hz in 30 second intervals). Multiple cycles of milling were sometimes required to achieve complete pulverization of the tissue to a fine powder. Isolation of RNA was achieved using the following procedures. The milled tissue samples were homogenized in cryopreservation tubes using a Polytron with disposable rotostator probes. The tissue was homogenized in 750 to 1000 uL of 100% TRIzol. 100% Chloroform was added to the TRIzol/GITC lysate (1:5 ratio) to facilitate separation of the organic and aqueous components using the phaselock (Eppendorf) system. The aqueous supernatant was further purified using the Promega SV-96 total RNA kit, incorporating a DNase treatment during the procedure. Isolated total RNA samples were then assayed for quality (Agilent Bioanalyzer) and yield (Ribogreen) metrics prior to amplification.

### RNA amplification and hybridization

Samples were amplified and labeled using a custom automated version of the RT/IVT protocol and reagents provided by Affymetrix. Hybridization, labeling and scanning were completed following the manufacturer's recommendations (Affymetrix). Sample amplification, labeling, and microarray processing were performed by the Rosetta Inpharmatics Gene Expression Laboratory in Seattle, WA. The expression data has been deposited in GEO (GSE25097, http://www.ncbi.nlm.nih.gov/geo/).

### Independent Validation Data Sets

Four recent studies were employed as independent validation. The first study consisted of 23 HCC tumor tissues collected in Singapore and assayed on the Affymetrix GeneChip HU133, resulting in the identification of a 57-gene signature associated with tumor recurrence [4]. The second study employed formalin-fixed paraffin-embedded non-tumor (i.e., adjacent normal) liver tissues from 82 Japanese HCC patients. A 186-gene signature was derived from this study using an Illumina array containing 6000 human genes [9]. The third study involved an HCC metastases cohort from Asia (N = 115 normal tissues) in which an Incyte 9, 180-reporter two channel array was used to profile the normal liver tissues [13]. Finally, a China-Belgium study (sample size N = 90) of mostly tumor tissues was profiled on a Qiagen 70-mer two channel array [14].

### Statistical Analysis

#### Classification of patients into good and poor prognosis groups

Prediction of cancer prognosis was performed by using the clinicopathologic phenotypes recorded at the time of surgery. There are a number of statistical learning techniques able to serve as classifiers. However, many of these methods do not directly accommodate two-dimensional outcome (e.g. survival and DFS). Herein, application of a univariate parameter selection and multivariate Cox model classifier was employed [6,7]. Univariate Cox regression models were applied to search for clinicopathologic parameters associated with outcomes. All significant clinicopathologic parameters were included in a multivariate Cox model. The use of the model yields the relative hazard for each patient, which serves as the linear predictor for survival endpoints and further division of the patients into good and poor prognosis groups based on the linear predictor. This approach and its prediction power were assessed using a leave-one-out (LOO) procedure and an independent testing sample set also collected in Hong Kong [10].

#### Expression trait processing

The intensity of all gene array experiments were normalized together using RMA methods [15]. Afterwards, the intensity was adjusted for gender and age of the patients. To avoid the influence of outliers, we fit the robust linear model (rlm, M-estimation with Tukey's bisquare weights), and used the residuals as the gene expression trait in all following analysis.

#### Further Classification of good and poor prognosis groups using gene expression

We examined whether the clinicopathologic phenotypes that were recorded at the time of surgery might predict cancer prognosis. Again, we applied the univariate Cox model for feature selection and multivariate Cox model for classification/prediction. Importantly, the classifier should use both the clinicopathologic parameters and expression biomarkers. As discussed above, feature selection on the entire dataset firstly identified genes that associate with cancer stage, which are of significant scientific value. However, these genes may not improve the prediction performance since they carry information that is redundant with the clinicopathologic data. Further, in the good and poor prognosis groups defined by the clinicopathologic parameters, the gene signatures were likely to be different. Therefore, we conducted the feature selection in the two prognosis groups separately.

In brief, the univariate Cox regression models was applied to search for gene expression traits associated with outcomes. The top 100 genes were included in a multivariate Cox model as the prediction model. Given there are only slightly more than 110 patients in either the good or poor prognosis groups, a linear model with 100 regressors would be unstable. Therefore, we conducted a further reduction in dimensionality using principle component analysis (PCA), a common approach to handle gene expression data in prognosis predictions [7]. The primary motivation for this approach was the fact that the top 100 genes are correlated; therefore, the top principle components (PCs) are able to extract most of the information and only cost a few degrees of freedom in the linear model. The top 6 PCs were used to build the Cox model for prediction, since they on average explained 80% of the variation in the 100 genes. We also explored additional models varying the number of genes (e.g. 50 and 200) and number of PCs (e.g., 5), and the results were consistent. From the model, we derived the relative hazard for each patient, which serves as the linear predictor for survival endpoints [10]. Further, we divided the patients into two sub-groups using the linear predictor, and the performance of the prediction was assessed by a leave-one-out (LOO) procedure. In each LOO iteration, we reserved one patient for testing and used the remaining patients (say N-1 patients) for training. On these N-1 training patients, we ran the univariate Cox model on each gene and derived the pvalue for association between prognosis (e.g. survival) and the gene's expression value. We then picked 100 genes with the smallest pvalues. With the 100 genes over N-1 patients, we constructed the 6 PCs, which then served as independent variables in a multivariate Cox model. This model and its coefficients captured the association between HCC prognosis and the PCs. Next, we projected the gene expression values of the reserved patient on the PC space (defined on the training patients) to obtain the coordinates of the first 6 PCs. We plugged these 6 coordinates into the multivariate Cox model and calculated the relative hazard for the reserved testing patient. After N LOO iterations, we derived the relative hazard for every patient, which was actually the linear predictor. Lastly, we used the log-rank test and Kaplan-Meier plot to examine and visualize the performance of the linear predictor. This scheme is similar to a previous report using clinicopathologic parameters to predict HCC outcome [10], except for the extra step of PCA dimensionality reduction.

## Results

### Clinicopathologic parameters predict HCC prognosis

**Figure 1:**
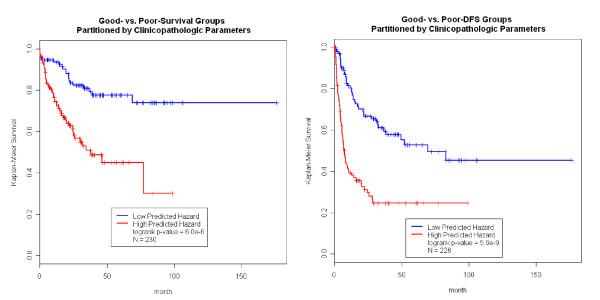
**Clinicopathologic Parameters Predict Survival and DFS on Patients with Available Normal Tissue Gene Expression Data**. In the left panels, the clinicopathologic parameters can classify the Hong Kong HCC patients into the good prognosis and poor prognosis groups that have distinct survival outcome. Similarly, in the right panels, we also classify the patients into two groups of distinct disease-free survival (DFS).

Tumor and adjacent non-tumor liver tissues were collected from 272 Chinese HCC patients who received curative surgery (referred to here as the HCC cohort). Additional File 1 Table S1 summarizes the characteristics of this cohort. Nearly 2/3 of the patients were right-censored (67.8%) and the other 1/3 (32.2%) of the patients were deceased (failure) upon data analysis. Half of the patients (51.1%) suffered from tumor recurrence during the follow-up period. The primary endpoints (overall survival and DFS) were found to be significantly associated with tumor size, AFP, ALBU, venous infiltration, pTNM and new AJCC stage, and NOTN. Previous analysis has shown that these clinicopathologic parameters can classify patients in the HCC cohort into two groups (denoted as good and poor prognosis groups) that give rise to distinct clinical outcomes [10]. For the survival endpoint, the good and poor survival groups contained roughly equal numbers of patients and were significantly different with respect to clinical outcome (log-rank test p-value of 6.0E-6; Figure 1 left panels). The linear predictor (*h*) shown in Figure 1 was derived using a leave-one-out (LOO) cross validation procedure to reduce biases that result from over fitting. Similarly, we classified the patients into good-DFS and poor-DFS groups (log-rank test p-value = 5.6E-9, Figure 1 right panel) [10]. Within each survival or DFS group, the clinicopathologic parameters could not further partition patients into subgroups of significantly different prognosis. In other words, the variation of prognosis within each group was not explained by clinicopathologic parameters.

### Combination of clinicopathologic and gene expressional biomarkers enhances prediction

To advance our understanding of the molecular processes and to extend the predictive power of the clinicopathologic variables, we explored the relationship of gene expression to outcome. Tumor and adjacent normal tissues were profiled using the Affymetrix whole genome expression array. The experiment surveyed 37, 585 unique transcripts, among which 23, 788 were mapped to well-annotated genes. Using the gene expression traits to predict group membership, we observed similar predictive power as was achieved using the clinicopathologic parameters alone.

**Figure 2:**
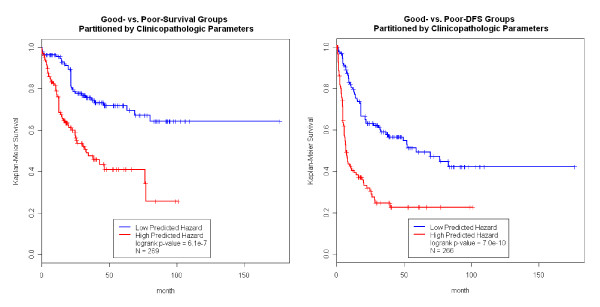
**Clinicopathologic Parameters Predict Survival and DFS on Patients with Available Tumor Tissue Gene Expression Data**. In the left panels, the clinicopathologic parameters can classify the Hong Kong HCC patients into the good prognosis and poor prognosis groups that have distinct survival outcome. Similarly, in the right panels, we also classify the patients into two groups of distinct disease-free survival (DFS).

In order to identify biomarkers that further enhance prognosis prediction based on the clinicopathologic parameters, a stratified analysis using univariate gene expression feature screening was carried out within each prognosis group (Figure 1&2). Under the null hypothesis the pvalues generated by this screening should follow a uniform distribution, substantial enrichment of low pvalues indicated a large number of prognosis-associated genes that might offer predictive power, shown Additional File 2 Figure S1. However, many of the prognosis-associated genes captured the same information as the clinical parameters, and contributed no extra prediction power. In the stratified analysis, we were able to identify genes associated with survival or DFS in each prognosis group. For example, many genes in the tumor tissue were associated with survival in the good-survival group but not in the poor-survival group (Additional File 3 Figure S2), suggesting that tumor gene expression may further enhance prognosis prediction in the good-survival group but not the poor-survival group. Interestingly, there were many normal tissue gene expression traits associated with DFS in the good DFS group, while many tumor tissue gene expression traits associated with DFS in the poor DFS groups (Additional File 3 Figure S2), again suggesting that the gene expression data may help stratify patients according to DFS beyond what could be achieved by the clinicopathologic parameters alone.

**Figure 3:**
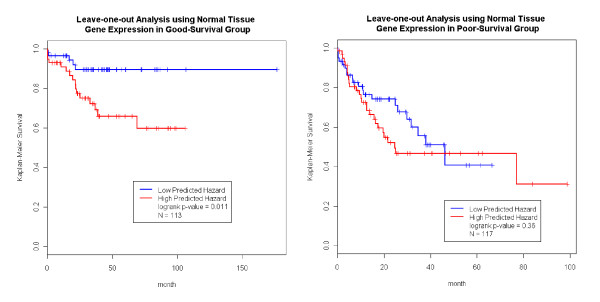
**Normal Tissue Expression Predicts Survival**. Within the good prognosis and the poor prognosis groups, clinicopathologic parameters could no longer separate patients into partitions of different survival outcome. Meanwhile, adjacent normal tissue gene expression provided extra information to further refine the prognosis prediction. Using a LOO framework with the dimension reduction and multivariate Cox model, we assigned predicted hazard for each patient. The hazard was able to further separate the good prognosis group, but not the poor prognosis group.

Motivated by these results, we combined the gene expression data with the clinicopathologic parameters to construct models to predict HCC prognosis and compare to those models constructed from the clinicopathologic data alone. We again employed a LOO cross validation strategy in each subset of patients to minimize over-fitting. For example, in the good-survival group, the LOO procedure was performed on the 113 patients with available normal tissue gene expression data (Figure 3, left panel).

**Figure 4:**
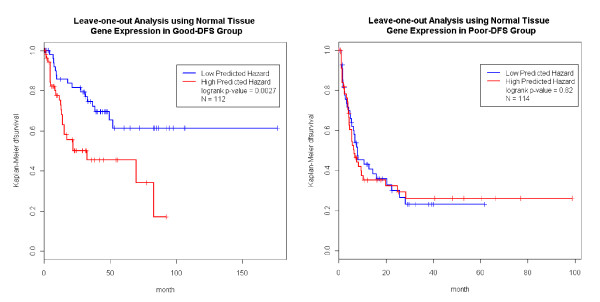
**Normal Tissue Expression Predicts DFS**. Using adjacent normal tissue gene expression data, we derived the predicted hazard within the good and poor DFS groups. The predictor (predicted hazard) further separated the patients in the good prognosis group, but not in the poor prognosis group.

The normal tissue expression data improved the prediction for the good-survival group (p-value = 0.011, Figure 3 left panel). The blue line in Figure 3 represented an excellent survival function (over 90%) for a group of patients carrying both favorable clinicopathologic and gene expression profiles. The same procedure was conducted on the 117 patients in the poor-survival group with available normal tissue gene expression data. As expected, the expression data could not further improve the predictive power of the model (Figure 3, right panel). Prediction in the good-DFS (p-value = 0.0027), but not the poor-DFS group was also further improved by incorporating normal tissue expression data into the model (Figure 4).

**Figure 5:**
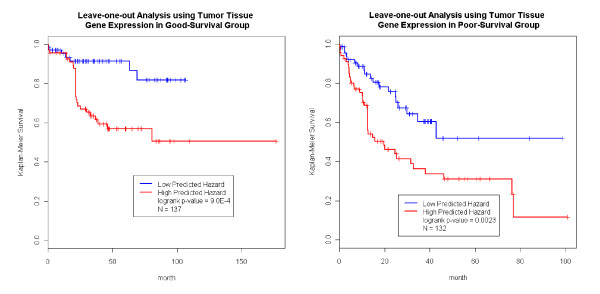
**Tumor Tissue Expression Predicts Survival**. Using tumor tissue gene expression profile, we obtained the predicted hazard within the good survival and poor survival groups. The predictor further separated the patients in both the good survival and the poor survival groups.

**Figure 6:**
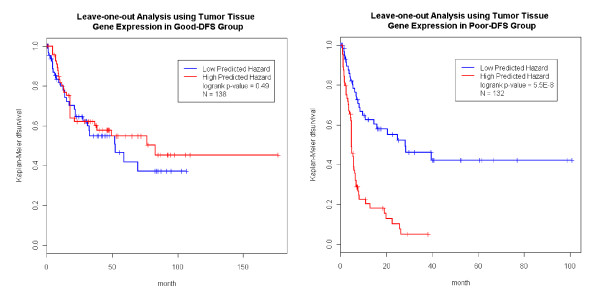
**Tumor Tissue Expression Predicts DFS**. Using tumor tissue gene expression profile, we obtained the predicted hazard within the good DFS and poor DFS groups. The predictor further separated the patients in poor DFS group, but not in the good DFS groups.

We carried out the same analysis just described using the tumor tissue expression data. It was noted that the sample sizes of the tumor tissue analysis was different from that of normal tissue (Figure 1&2). Figure 5 shows that the prediction in the good-survival (p value = 9.0E-4) and poor-survival (p-value = 0.0023) groups were further improved by the incorporation of the tumor expression data. Curiously, the pattern was opposite for DFS compared to what we observed in the normal tissues. For the poor-DFS group, tumor expression significantly further partitioned patients into good and poor DFS subgroups (p-value = 5.5E-8; Figure 6, right panel). No prediction improvement for the good-DFS group was observed. Since both normal and tumor tissues had predictive power in the good-survival group (Figure 3 and 5), we were motivated to explore predictive models incorporating expression traits from both tissue types. The number of patients with both tissues available was N = 110. The combined data resulted in a further improvement of the prediction performance (Additional File 4 Figure S3).

### Independent validation using published HCC prognosis gene signatures

The identification of prognosis-associated gene signatures in HCC is an active field of research, with previous reports indicating that different gene signatures may be harvested from tumor and adjacent non-tumor tissues [4,9,13,14,16]. To capture information from the expression data that was not yet covered by the clinicopathology, the clinicopathologic parameters should be conditioned on during the search for expression signatures, as we have done. Unfortunately, many of the reported studies did not follow such a strategy. Ideally, our gene signatures would be tested in independent HCC cohorts. However, raw clinical outcome data was not available for any of the published studies [4,9,13,14]. Therefore, we compared our gene signature, which showed predictive value towards HCC prognosis, with published signatures. We would not expect perfect concordance among the signatures given the different patient recruitment criteria, experimental conditions, array platforms, and statistical methods across the studies. For example, five different array platforms were used in studies listed in Table 1 and 2: Affymetrix whole genome custom array (current study), Illumina 6000-gene human array [9], Qiagen 70-mer two channel array [14], Incyte 9, 180-reporter two channel array [13], and Affymetrix GeneChip HU133A&B arrays [4]. More importantly, since the sample sizes for the published studies were all small (n < 200), the power to capture prognosis-associated genes was low. Hence, we applied liberal p-value cutoffs (e.g. 5E-4) in order to capture a majority of the HCC prognosis-associated genes and in turn enhance the comparison to the published signatures (Table 1, 2 and Additional File 5 Table S2). The survival- and DFS-signatures we identified significantly overlapped with all published gene lists (Table 1). For example, Lee et al reported a comprehensive HCC gene signature on a Chinese-Belgium dataset that overlapped our results in a highly significant manner (4.8 fold enrichment; Fisher Exact Test p-value = 1.4E-12 for the tumor tissue DFS signature, Table 1 and 2). As one would expect, when the study design of a published gene signature matches our design, we observed a more extensive overlap with the signatures. For example, Hoshida et al. [9] and Budhu et al. [13] studied adjacent non-tumor liver tissues in HCC patients. Their gene lists overlapped with our normal tissue signature more significantly than with the tumor signature. Alternatively, Lee et al. reported a gene list that overlapped heavily with our tumor tissue signatures. It is noteworthy that the hypergeometric test p-values were driven by both magnitudes of overlaps and the size of the published gene lists. The overlap among the various gene lists were summarized in Additional File 6, 7, 8 and 9, Table S3. The first part of Additional File 5, 6, 7, and 8, Table S3 highlighted genes that reached genome-wide significance in our HCC data. There was no adjustment for clinicopathologic parameters in order to retain consistency with other published signatures. A strict cutoff (Cox p-value ≤ 2e-6) was applied to the identified genes that were genome-wide significant after a Bonferroni correction. The second part of Additional File 6, 7, 8 and 9, Table S3 summarized genes that appeared in at least two other gene lists.

**Table 1:** Using Independent HCC Studies to Validate HKU Gene Signature Identified at 0.0005 Level*

HKU Gene Signature in Normal Tissue		
	Survival (1074 HKU Gene Signature) ^‡^	
	Overlapping Genes	Enrichment p-value
Japanese Gene Signature	33	4.8E-13
Chinese-Belgium Gene Signature	41	1.1E-13
Asia HCC Metastases Gene Signature	31	1.0E-5
Singapore Gene Signature	5	1.2E-2
	
	**Disease Free Survival **(1195 HKU Gene Signature)	
	Overlapping Genes	Enrichment p-value
Japanese Gene Signature	34	1.9E-12
Chinese-Belgium Gene Signature	36	2.7E-9
Asia HCC Metastases Gene Signature	36	8.1E-7
Singapore Gene Signature	5	1.9E-2

**HKU Gene Signature in Tumor Tissue**		
	**Survival **(660 HKU Gene Signature)	
	Overlapping Genes	Enrichment p-value
Japanese Gene Signature	5	0.36
Chinese-Belgium Gene Signature	22	4.6E-7
Asia HCC Metastases Gene Signature	6	0.13
Singapore Gene Signature	1	0.11
	
	**Disease Free Survival **(2169 HKU Gene Signature)	
	Overlapping Genes	Enrichment p-value
Japanese Gene Signature	6	0.12
Chinese-Belgium Gene Signature	28	1.4E-12
Asia HCC Metastases Gene Signature	20	1.6E-5
Singapore Gene Signature	1	0.27

*Herein, we compared gene signatures obtained in following studies: HKU study (sample size N = 229 for adjacent normal tissues and N = 267 for tumor tissues), Japan study (175 gene signature based on sample size N = 82), Asia HCC Metastases study (307 gene signature based on sample size N = 115), China-Belgium study (247 gene signature based on sample size N = 90) and Singapore study (43 gene signature based on sample size N = 23).

^‡^We selected genes associated with survival or disease-free survival using nominal p-value of 0.01.

**Table 2:** Using Independent HCC Studies to Validate HKU Gene Signature Identified at 0.05 Level

HKU Gene Signature in Normal Tissue		
	Survival (7400 HKU Gene Signature) ^‡^	
	Overlapping Genes	Enrichment p-value
Japanese Gene Signature	92	1.1E-9
Chinese-Belgium Gene Signature	133	3.1E-14
Asia HCC Metastases Gene Signature	144	2.0E-9
Singapore Gene Signature	19	2.4E-2
	
	**Disease Free Survival **(7661 HKU Gene Signature)	
	Overlapping Genes	Enrichment p-value
Japanese Gene Signature	96	1.8E-10
Chinese-Belgium Gene Signature	131	3.6E-12
Asia HCC Metastases Gene Signature	145	1.3E-8
Singapore Gene Signature	21	7.6E-3

**HKU Gene Signature in Tumor Tissue **		
	**Survival **(5403 HKU Gene Signature)	
	Overlapping Genes	Enrichment p-value
Japanese Gene Signature	66	2.4E-6
Chinese-Belgium Gene Signature	142	5.5E-33
Asia HCC Metastases Gene Signature	97	1.2E-4
Singapore Gene Signature	19	4.9E-4
	
	**Disease Free Survival **(4377 HKU Gene Signature)	
	Overlapping Genes	Enrichment p-value
Japanese Gene Signature	58	9.1E-7
Chinese-Belgium Gene Signature	131	1.7E-35
Asia HCC Metastases Gene Signature	89	1.9E-6
Singapore Gene Signature	15	2.8E-3

^‡^We selected genes associated with survival or disease-free survival using nominal p-value of 0.05.

### eSNPs underlying differentially connected genes linked to HCC prognosis

To explore the predictive genes in the context of a normal-to-tumor network reconfiguration, we looked at whether the genetic perturbations of the predictive genes were associated with HCC prognosis. Two sets of genes were explored in this way: 1) genes differentially connected between the normal and tumor tissue co-expression networks (gene-set-1), as detailed by Lamb et al [17]; and 2) genes whose expression levels were significantly associated with copy number abnormalities (CAN) in the tumor tissue (gene-set-2).

To explore whether these gene sets were enriched for eSNPs that associate with HCC prognosis, we genotyped DNA isolated from adjacent normal tissues using the Illumina 650Y SNP arrays and characterized the genetic architecture of gene expression based on a previously described method [18]. This genome-wide association study of gene expression resulted in 1, 296 *cis *expression QTLs (eQTLs) [18] at a 10% FDR (translating to p-value cutoff = 9E-6). We termed the significant SNPs (associated with the corresponding expression trait at a p < 9E-6) underlying the eQTLs as eSNPs. There were 707 and 2, 840 eSNPs for gene-set-1 and gene-set-2, respectively. Further, we examined if the eSNPs linked to gene-set-1 and -2 were enriched for SNPs that associated with clinical endpoints (referred to as clinical SNPs or cSNPs). In brief, the cSNPs were identified using Cox models focusing on survival or DFS. The model was adjusted for clinicopathologic parameters.

At an α level of 0.01, we identified 6, 399 cSNPs associated with HCC survival (referred to as survival-cSNPs). Interestingly, the eSNPs underlying gene-set-1 were 2.1-fold enriched for survival-cSNPs (p = 7E-4); and the eSNPs underlying gene-set-2 were 1.7-fold enriched for survival-cSNPs (p = 2E-5). In parallel, at an α level of 0.01, the SNP screening using a Cox model yielded 5, 246 SNPs associated with DFS (referred to as DFS-cSNPs). We found the eSNPs underlying gene-set-1 were not enriched for DFS cSNPS; in contrast, eSNPs for gene-set 2 were 1.9-fold enriched for DFS cSNPs (p-value = 5E-7). For comparison, we randomized the clinical endpoints and repeated the Cox modeling, and at α level of 0.01, we yielded pseudo-cSNPs, which arose purely from type I errors. The eSNPs underlying gene-set-1 and -2 were not enriched in the pseudo-cSNPs.

## Discussion

Numerous studies have reported the ability of clinicopathologic parameters [2,3,10,19] and gene expression traits [4,9,13,14] to predict HCC prognosis. However, the sample sizes of these previous studies were small, and there were no systematic efforts to compare the performance of these two types of predictors or combine them in one unified model. As we previously showed, several clinicopathologic parameters that are easily and routinely measured, provided excellent predictive power for outcome in HCC [10] and result in predictions that were readily applicable to clinical practice. Given their utility, it is natural to attempt to further enhance the clinicopathology-based prediction model by adding gene expression data. We conducted a head-to-head performance comparison between gene expression predictors derived from normal and tumor tissue (denoted as *h_gene-expression_*) vs. predictors derived solely from clinicopathology (*h_pathology_*; Materials and Methods) and benchmarked them in a LOO framework (Figure 1, 2 and Additional File 10 Figure S4). Please note, the genes used in the prediction models might be different with regard to normal vs. tumor tissue expression, as well as in each LOO iteration. Overall, *h_gene-expression _*and *h_pathology _*performed similarly. The *h_gene-expression _*of tumor tissue was better than *h_pathology _*in predicting DFS, but *h_gene-expression _*slightly underperformed *h_pathology _*in all other scenarios. Overall, gene expression was not superior to clinicopathology in predicting prognosis. One reason might be that gene selection primarily identified genes correlated with clinicopathologic parameters (e.g. cancer stage). To assess if expression variables could be identified that enhance predictive power, stratified analysis and computed *h_gene-expression _*within the good- and poor subgroups that go beyond the clinicopathologic parameters were performed. A combination of these two types of data resulted in the identification of a group of patients with near perfect survival after surgery (blue curve, left panel, Figure 3). These patients had both favorable clinicopathologic and gene expression profiles (they enjoyed a 90% survival rate over 100 months). In contrast, we found that DFS over 30 months for patients with both poor clinicopathologic and gene expression profiles whose was lower than 10%.

The focus of this study is stratified modeling, which is a natural extension of our previous work. Alternatively, we can build a single model incorporating clinicopathologic parameters and gene expression data simultaneously (Additional File 11 Figure S5). The prediction framework is identical to the above analyses except the multivariate Cox model included both the clinicopathologic parameters and the top 6 PCs. In the gene selection step we also included clinicopathologic parameters in the Cox model and then picked 100 genes with the smallest pvalues. The overall prediction was better than using gene expression alone (Additional File 10 Figure S4), indicating clinicopathology captured valuable information beyond gene expression. However, comparing to Figure 1 and 2, adding gene expression only enhanced prediction in one scenario (tumor gene expression improved the prediction of survival). A possible explanation would be that different gene sets were associated with prognosis across the strata defined by clinicopathology, and these gene sets offer various prediction value. For example, shown in Figure 2, normal tissue expression was used in prediction in the good-survival stratum but not the poor-survival stratum. In the single model approach, genes with little prediction value also entered the model, bringing noise and reducing the performance. Lastly, we also evaluated a single model incorporating clinicopathologic parameters, and expression profiles of both normal and tumor tissue (Additional File 12 Figure S6). We reduced the expression profile of each tissue to 6 PCs, therefore, a total of 12 PCs entered the model. Overall, such models did not greatly outperform the models based on clinicopathology alone (Figure 1&2) or models based on clinicopathology and expression profiles (Additional File 11 Figure S5). Again, this lack of improvement could be attributable to noises introduced into the prediction.

HCC tumor tissue and adjacent non-tumor tissues harbor distinct prognosis-associated signatures and lead to differences in predictive power. Importantly, we noted that the gene signature derived from the tissues significantly overlapped. Consistent findings were also reported on Chinese and Belgium HCC patients [14], Asian [13] and Singapore patients [4]. However, for 82 Japanese patients, Hoshida et al found gene-expression profiles of tumor tissue failed to yield a significant association with survival [9]. This result is inconsistent with the fact that gene expression traits in tumor tissues were correlated with cancer stage, and cancer stage was strongly associated with survival [10]. The failure to detect gene expression traits with predictive power in this instance could be due to the small sample size (N = 82) and the use of formalin-fixed, paraffin-embedded tissues [9].

The mechanistic basis whereby gene expression traits predict the aggressiveness of a tumor remains to be defined. One of the striking features of this analysis and others [4,9,13] was the finding that signatures in normal tissue adjacent to the tumor is highly predictive of prognosis. It had been suggested that mechanistically these signatures represented a so called "field-effect" capturing damage to liver tissue and the state of inflammation related to the likelihood of subsequent tumors arising [9,20]. In other words, the field effect hypothesis implied that the signatures did not relate directly to processes in the tumors per se but rather the environment from which tumors might arise. An alternate hypothesis supported by our results was that the signatures are mechanistically connected to tumor specific events in some way, given the genes associated with survival (p < 2e-159, fold enrichment 31.01) and DFS (p < 5e-152, fold 29.31) in adjacent normal and in tumor tissues significantly overlap.

To address whether the "field effect" or this alternate hypothesis was better supported by the data, we first examined the evidence that tumors directly affect the surrounding normal tissues via secreted factors. If that were the case we would expect that the adjacent normal gene expression patterns would be correlated with DNA copy number abnormalities (CNA) in the tumor tissue, given we previously showed CNA was strongly connected to tumor gene expression [10]. No significant associations beyond what would be expected by chance were found. We were also able to exclude significant invasion of tumor cells into the adjacent normal tissues given we observed no significant associations between normal tissue derived CNA and normal tissue gene expression [10].

Given this result, how might signatures in normal tissues mechanistically relate to tumor events? Herein, we took advantage of previous work on this dataset. We described the massive gene expression network changes that occur during HCC tumorigenesis, and such rearrangements were likely driven by tumor CNA [10]. In brief, we defined gene pairs where the pair was significantly correlated in one setting and significantly less correlated in another [10]. Using stringent cut-offs, there were 8, 736 genes differentially connected between the adjacent normal and tumor tissues with ~86% of cases representing a loss of connectivity in the tumor (LOC) and the remaining ~14% representing a gain of connectivity (GOC) [10]. We therefore tested whether the predictive gene expression traits from the adjacent normal tissue were enriched for differentially connected genes and found that indeed they were. The genes associated with survival in adjacent normal tissue (Cox p < 0.01, Additional File 6, 7, 8 and 9, Table S3) were enriched for genes participating in differential connections (p < 1.98e-80, fold enrichment 2.69). Similarly gene expression traits in adjacent normal tissue associated with DFS (Cox p < 0.01, Additional File 6, 7, 8 and 9, Table S3) were enriched for genes identified as differentially connected in the tumor tissue (p < 5.2e-69, fold 2.52).

Our previous reports also documented that a large fraction of expression variation can be explained by CNA in the tumor tissues [10]. Therefore, we asked if the predictive genes (Additional File 6, 7, 8 and 9, Table S3) were enriched for genes that associated with *cis*-acting CNAs in tumors. We found that both survival- and DFS-associated genes in normal tissue were enriched for genes associated with CNAs in *cis *in the tumor tissue (p-values were 2.99e-8 and 1.72e-11, respectively). Since genes in adjacent normal tissues were measured entirely separately from genes in the tumor, there was no a priori reason for them to behave similarly unless there was a mechanistic connection. We found that predictive genes from adjacent normal tissue were selectively enriched in network re-arrangements and enriched for genes that associate with CNA associated tumorigenesis, strongly suggesting that these genes represent important functions targeted for alteration in tumors. Stated in another way, the predictive signatures in adjacent normal tissue are a measure of the ability of the tissue to alter expression networks to enter a more aggressive state.

If the predictive genes were the determinant of the normal-to-tumor network reconfiguration, then we would expect that genetic perturbations of these genes would also be associated with HCC prognosis. We examined the normal liver tissue eSNPs underlying two sets of genes (1) genes that are differentially connected between the normal and tumor states in the HCC cohort, and (2) genes whose expression levels were significantly explained by CNA in the liver tumor tissue. We found that the eSNPs controlling the expression level of these two sets of genes were enriched for association with HCC survival and DFS. This result supports the hypothesis that the mechanisms by which genes in normal tissue are predictive of prognosis reflects their ability to facilitate the transition from a normal tissue network to a tumor network, where this transition determines cancer progression.

We have demonstrated the excellent predictive power of our approach by combining clinicopathologic parameters and gene expression profiles. As a result, we expect that this approach to provide valuable guidance for HCC treatment/management in clinical practice. More importantly, based on our previous work on the architecture of coexpression networks in adjacent normal and tumor tissues [10], we proposed a general mechanism of how predictive genes influence HCC prognosis. The massive rearrangement of expression networks plays a central role in HCC progression, which was reflected in the ability of such genes to predict HCC prognosis. This also explained why the predictive genes significantly overlapped between the adjacent normal and tumor tissues, since such genes would ostensibly continue to reflect the ongoing alterations in network state.

## Conclusions

Hepatocellular carcinoma (HCC) is highly lethal, and its prognosis following surgery varies substantially, in a manner which is yet to be explained. Our contributions to understanding the progression of HCC are three fold. (1) We established a comprehensive algorithm to incorporate clinicopathologic and gene expression parameters, which greatly improves the prediction of HCC prognosis. (2) Using a large sample size, we characterize the gene expression alternation in HCC tumor and adjacent normal tissues, and their association to overall survival and disease-free survival. (3) We proposed a general framework explaining why gene expression in both tumor and adjacent normal tissues can predict HCC prognosis. In brief, the gene expression networks undergo massive a transition during tumorigenesis and tumor progression, where normal tissue gene networks are destroyed and tumor gene networks are establish. States of the genes in these key networks determine the likelihood and rate of such massive transition as well as tumor progression and as a result the expression levels of these genes predict HCC prognosis.

## Declaration of competing interests

The authors declare that they have no competing interests.

## Authors' contributions

All authors read and approved the final manuscript. KH, all figures and tables, experimental design and manuscript preparations. JL, MM, DG, MDF, CM EES, HD and JML, experimental design. CZ, processing the mRNA microarray readings and calculation of the expression values. TX and EC, processing the SNP arrays and calculation of the CNV values. KW, BZ and HZ, construction of the expression networks. NPYL, ION, PCS, RTPP, and JML, collection of tissue samples and clinicopathologic parameters.

## Pre-publication history

The pre-publication history for this paper can be accessed here:

http://www.biomedcentral.com/1471-2407/11/481/prepub

## Supplementary Material

Additional file 1 **Supplementary**. Supplementary Table 1 Click here for file

Additional file 2 **Figure S1. Crude Pvalues of Association between HCC Prognosis and Gene Expression Profiles**. Histogram of p-values of the univariate search for genes associated with survival outcome. The substantial enrichment for small p-values indicated potential predictive power of the gene expression data. Click here for file

Additional file 3 **Figure S2. Association between HCC Prognosis and Gene Expression Profiles in Strata Defined by Clinicopathologic Parameters**. Histogram of p-values of the univariate search for genes associated with HCC outcome, conducted within good and poor prognosis strata. Click here for file

Additional file 4 **Figure S3. Improving prediction in the good-survival stratum using normal tissue and tumor tissue gene expression profiles together**. Shown in Figure 2 and 4, both normal tissue and tumor tissue had prediction value in the good-survival group. Restricted in the patients with both normal and tumor tissues available (N = 110), we followed LOO procedure and derived h incorporating expression data of both tissue. The prediction performance was further improved. Presented in the blue curve (lower right panel), we identified 55 patients with excellent clinicopathologic and gene expressional profiles (both normal and tumor tissues). Their 100 months survival was above 95%. Click here for file

Additional file 5 **Supplementary**. Supplementary Table 2 Click here for file

Additional file 6 **We integrated the Hong Kong gene list with published HCC gene signatures and create the list of HCC prognosis-associated genes**. The first part of the list contains genes that reached genome-wide significance level in our Hong Kong data. Herein, we did not adjust for clinicopathologic parameter in order to remain comparable to published signatures. Strict cutoff (Cox p-value ≤ 2e-6) were applied to our results, therefore, the identified genes were genome-wide significant after Bonferroni correction. The second part of the list contains genes that appeared at least twice in HCC gene lists. Here, we applied a liberal p-value cutoff (Cox p-value ≤ 0.01) to Hong Kong data. The second column of the table presents the Wald's test p-values, where "-" denotes the p-value above 0.01. The columns 3-6 are indicators for published HCC signatures, where "1" denotes present and "-" denotes absent. Click here for file

Additional file 7 Supplementary Table 3B Click here for file

Additional file 8 Supplementary Table 3C Click here for file

Additional file 9 Supplementary Table 3D Click here for file

Additional file 10 **Figure S4**. Predicting HCC Prognosis using Gene Expression Profiles of Normal of Tumor Tissue Click here for file

Additional file 11 **Figure S5**. Predicting HCC Prognosis using Clinicopathologic Parameters + Gene Expression Profiles of Normal or Tumor Tissue Click here for file

Additional file 12 **Figure S6**. Predicting HCC Prognosis using Clinicopathologic Parameters + Gene Expression Profiles of Both Normal and Tumor Tissue Click here for file
