# Supplementary material for: Clinicopathologic and gene expression parameters predict liver cancer prognosis
Source: BMC Cancer. 2011 Nov 9;11:481. doi: 10.1186/1471-2407-11-481 (PMC3240666; doi:10.1186/1471-2407-11-481)
Supplement: Additional file 1 — Supplementary. Supplementary Table 1 [file 1471-2407-11-481-S1.PDF]

Table S1, Demographic and clinic characteristics of HCC patients in the initial training set

Variable Name	Mean±SD or %	Variable Name	Percentage
**Survival (month)**	33.8 ± 29.4	**Event**	
**Disease free survival (month)**	25.0 ± 28.9	Deceased	32.2
**Age (year)**	56.0 ± 12.0	Censored	67.8
**Male**	80.4%	**Child's grade**	
**Liver Function Parameters**		A	97.3
AFP [$\log_{10}$] (ng/mL)	2.18 ± 1.39	B	2.7
SGPT (U/L)	61.0 ± 51.4	**Family History of HCC**	20.7
SGOT (U/L)	64.3 ± 53.9	**Smoking**	
BILIRUBIN (μM)	14.5 ± 11.2	No	55.6
ALBUMIN (mg/mL)	40.2 ± 4.7	Moderate	30.5
		Heavy	13.9
**Tumor Size (cm)**	7.6 ± 4.1	**Alcohol Drinking**	
**Tumor Recurrence**	51.1%	No	60.5
**Venous Infiltration**		Moderate	23.3
Absence	50.6%	Heavy	16.2
Presence	49.4%	**Tumor Nodule (N =)**	
**Non-tumorous liver histology**		1	76.3
Cirrhotic	57.1%	2	6.8
Non-cirrhotic	13.9%	3	1.5
Chronic hepatitis	28.9%	4	1.1
**TNM Stage**		5	0.4
I	3.0%	6	0.8
II	41.1%	Multiple >6	13.2
IIIA	35.5%	**Edmondson Grade**	
IV	20.3%	Undifferentiated	1.3
**AJCC Stage**		Poorly Differentiated	18.3
I	41.5%	Moderate Differentiated	59.2
II	27.9%	Well Differentiated	21.3
IIIA	21.9%	**HBsAg Status**	
IIIB	7.5%	Positive	86.1
IV	1.1%	Negative	13.9
