# Supplementary figures and images for: Clinicopathologic and gene expression parameters predict liver cancer prognosis
Source: BMC Cancer. 2011 Nov 9;11:481. doi: 10.1186/1471-2407-11-481 (PMC3240666; doi:10.1186/1471-2407-11-481)

Figure S1, Crude Pvalues of Association between HCC Prognosis and Gene Expression Profiles

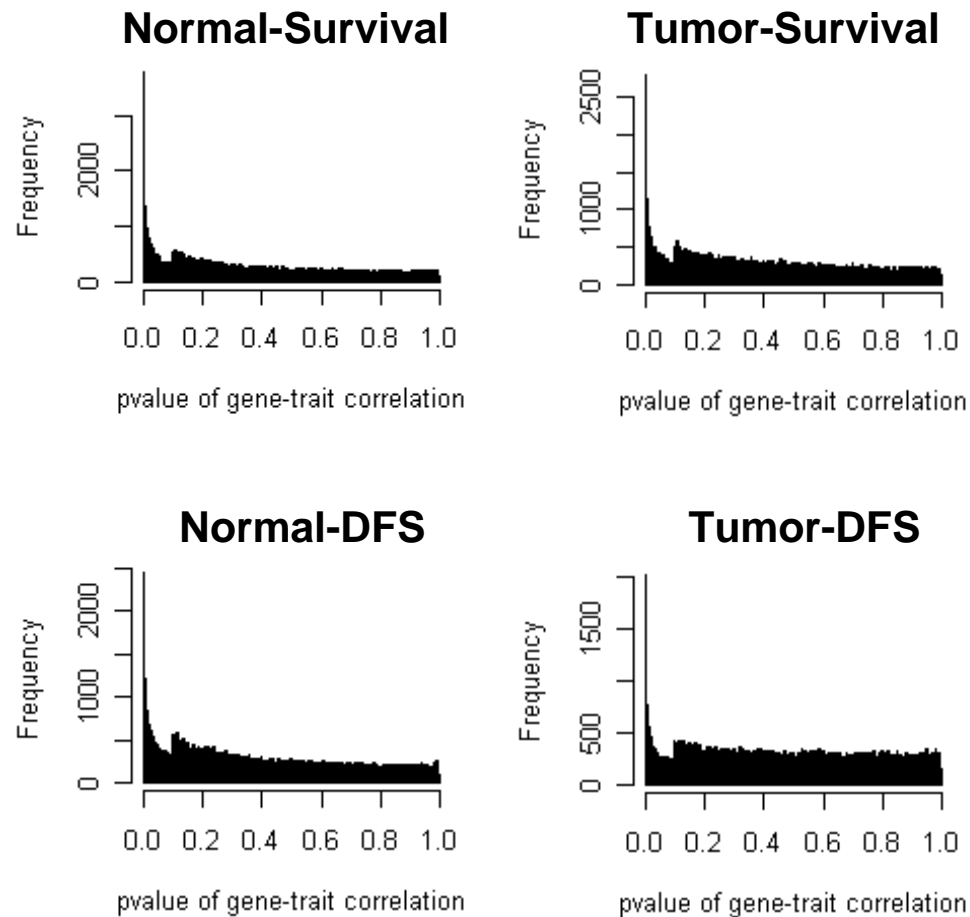

Supplement: Additional file 2 — Figure S1. Crude Pvalues of Association between HCC Prognosis and Gene Expression Profiles. Histogram of p-values of the univariate search for genes associated with survival outcome. The substantial enrichment for small p-values indicated potential predictive power of the gene expression data. [file 1471-2407-11-481-S2.PDF]

Figure S4, Predicting HCC Prognosis using Gene Expression Profiles of Normal or Tumor Tissues

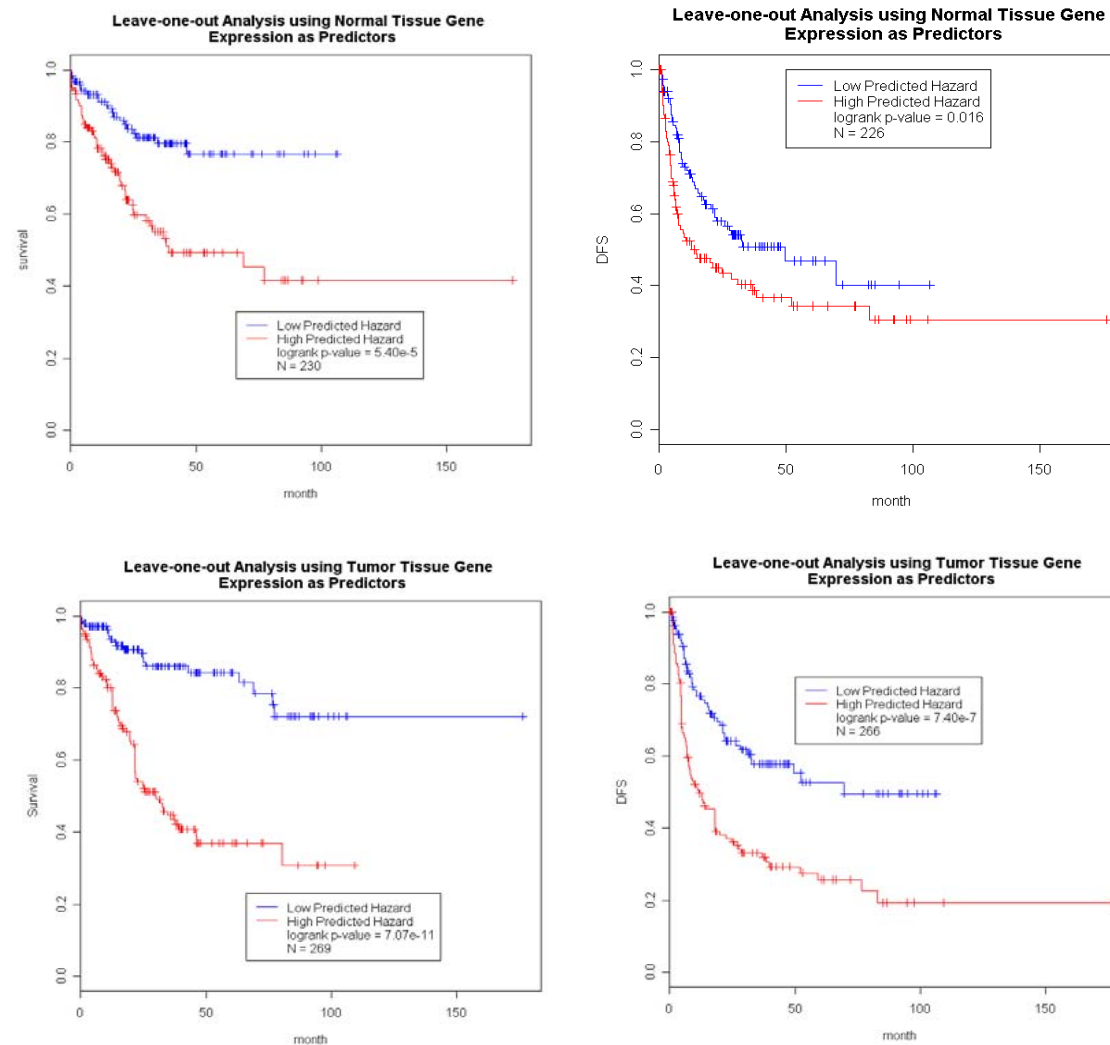

Supplement: Additional file 10 — Figure S4. Predicting HCC Prognosis using Gene Expression Profiles of Normal of Tumor Tissue [file 1471-2407-11-481-S10.PDF]
