# Supplementary material for: Clinicopathologic and gene expression parameters predict liver cancer prognosis
Source: BMC Cancer. 2011 Nov 9;11:481. doi: 10.1186/1471-2407-11-481 (PMC3240666; doi:10.1186/1471-2407-11-481)

# Figure S2, Association between HCC Prognosis and Gene Expression Profiles in Strata Defined by Clinicopathologic Parameters

## Figure S2A, Survival Endpoints

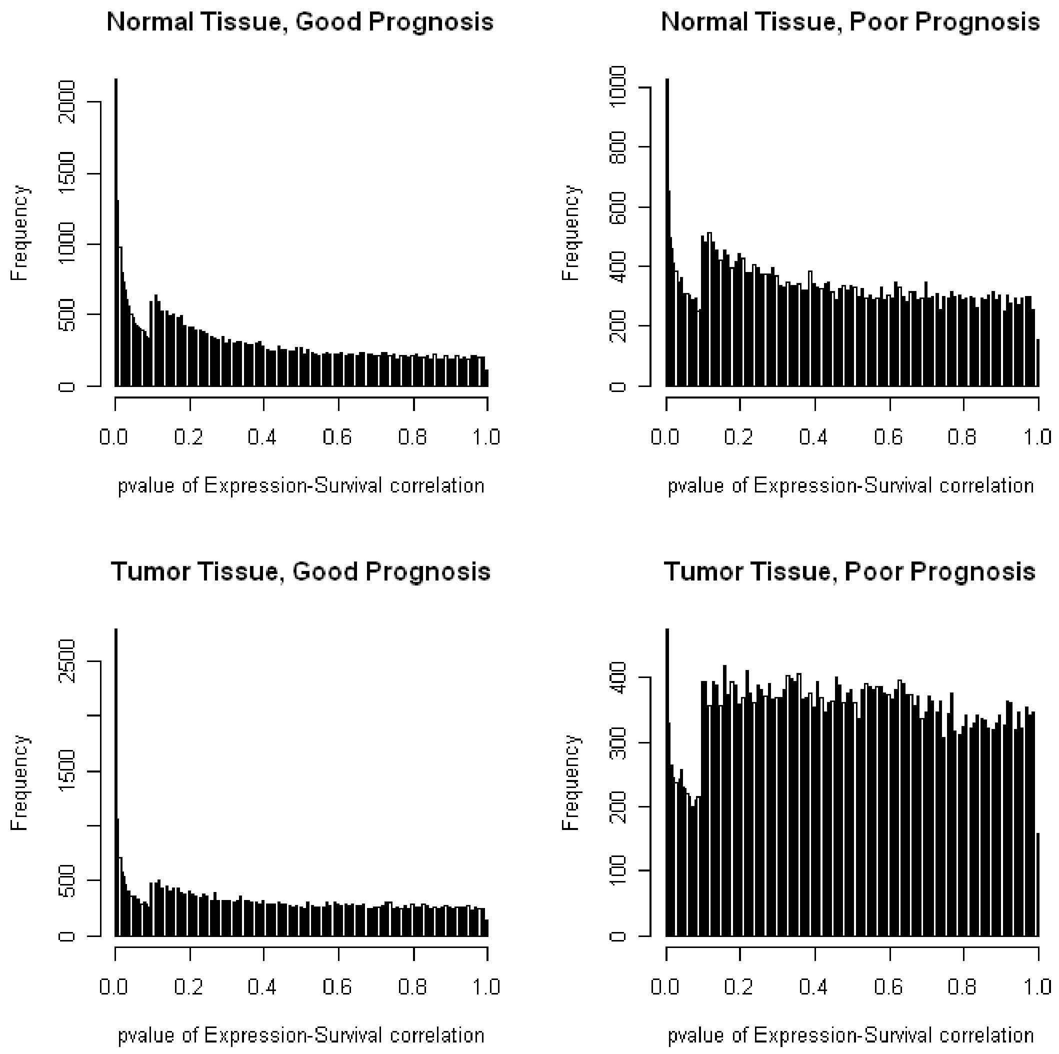

Figure S2B, Disease-Free Survival Endpoints

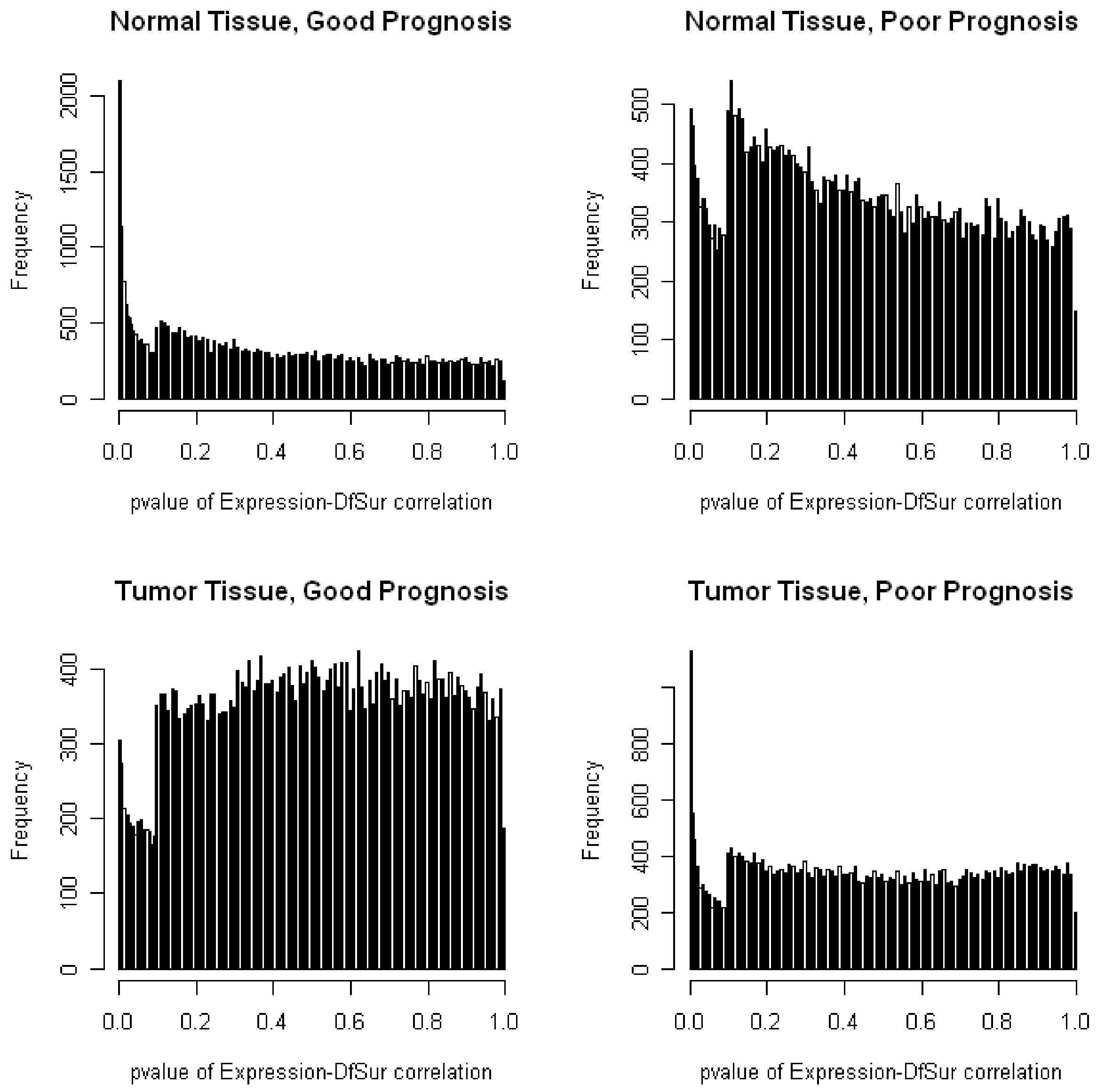

Supplement: Additional file 3 — Figure S2. Association between HCC Prognosis and Gene Expression Profiles in Strata Defined by Clinicopathologic Parameters. Histogram of p-values of the univariate search for genes associated with HCC outcome, conducted within good and poor prognosis strata. [file 1471-2407-11-481-S3.PDF]
