# Supplementary material for: Clinicopathologic and gene expression parameters predict liver cancer prognosis
Source: BMC Cancer. 2011 Nov 9;11:481. doi: 10.1186/1471-2407-11-481 (PMC3240666; doi:10.1186/1471-2407-11-481)

Figure S3, Improving prediction in the good-survival stratum using normal tissue and tumor tissue gene expression profiles

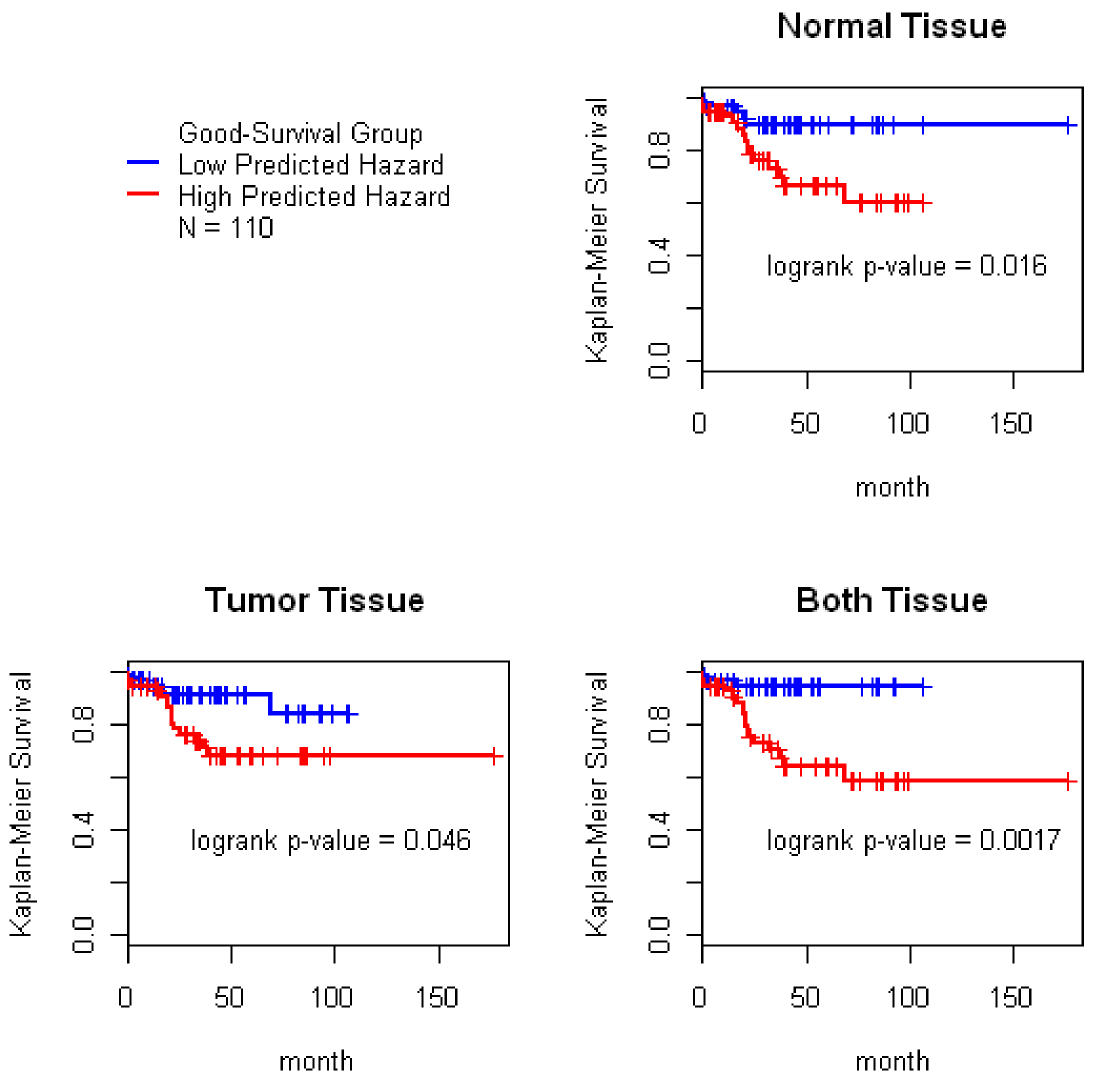

Supplement: Additional file 4 — Figure S3. Improving prediction in the good-survival stratum using normal tissue and tumor tissue gene expression profiles together. Shown in Figure 2 and 4, both normal tissue and tumor tissue had prediction value in the good-survival group. Restricted in the patients with both normal and tumor tissues available (N = 110), we followed LOO procedure and derived h incorporating expression data of both tissue. The prediction performance was further improved. Presented in the blue curve (lower right panel), we identified 55 patients with excellent clinicopathologic and gene expressional profiles (both normal and tumor tissues). Their 100 months survival was above 95%. [file 1471-2407-11-481-S4.PDF]
