# Supplementary material for: Clinicopathologic and gene expression parameters predict liver cancer prognosis
Source: BMC Cancer. 2011 Nov 9;11:481. doi: 10.1186/1471-2407-11-481 (PMC3240666; doi:10.1186/1471-2407-11-481)
Supplement: Additional file 5 — Supplementary. Supplementary Table 2 [file 1471-2407-11-481-S5.PDF]

Table S2A, Using Independent HCC Studies to Validate HKU Gene Signature Identified at 0.0001 Level*

**HKU Gene Signature in Normal Tissue**		
	**Survival** (497 HKU Gene Signature) ‡	
	Overlapping Genes	Enrichment p-value
Japanese Gene Signature	22	2.4E-12
Chinese-Belgium Gene Signature	23	5.0E-10
Asia HCC Metastases Gene Signature	14	2.2E-3
Singapore Gene Signature	2	6.0E-2
	**Disease Free Survival** (531 HKU Gene Signature)	
	Overlapping Genes	Enrichment p-value
Japanese Gene Signature	17	8.4E-8
Chinese-Belgium Gene Signature	22	9.2E-9
Asia HCC Metastases Gene Signature	19	2.1E-5
Singapore Gene Signature	3	1.5E-2
**HKU Gene Signature in Tumor Tissue**		
	**Survival** (328 HKU Gene Signature)	
	Overlapping Genes	Enrichment p-value
Japanese Gene Signature	3	0.22
Chinese-Belgium Gene Signature	11	1.8E-4
Asia HCC Metastases Gene Signature	9	0.34
Singapore Gene Signature	3	3.1E-2
	**Disease Free Survival** (266 HKU Gene Signature)	
	Overlapping Genes	Enrichment p-value
Japanese Gene Signature	4	4.7E-2
Chinese-Belgium Gene Signature	10	1.2E-4
Asia HCC Metastases Gene Signature	11	2.1E-4
Singapore Gene Signature	1	0.08

*Herein, we compared gene signatures obtained in following studies: HKU study (sample size N = 229 for adjacent normal tissues and N = 267 for tumor tissues), Japan study (175 gene signature based on sample size N = 82), Asia HCC Metastases study (307 gene signature based on sample size N = 115), China-Belgium study (247 gene signature based on sample size N = 90) and Singapore study (43 gene signature based on sample size N = 23).

‡We selected genes associated with survival or disease-free survival using nominal p-value of 0.01.

Table S2B, Using Independent HCC Studies to Validate HKU Gene Signature Identified at 0.01 Level

**HKU Gene Signature in Normal Tissue**		
	**Survival** (3940 HKU Gene Signature) ‡	
	Overlapping Genes	Enrichment p-value
Japanese Gene Signature	73	6.8E-16
Chinese-Belgium Gene Signature	96	9.7E-18
Asia HCC Metastases Gene Signature	83	1.1E-6
Singapore Gene Signature	16	2.8E-4
	**Disease Free Survival** (4267 HKU Gene Signature)	
	Overlapping Genes	Enrichment p-value
Japanese Gene Signature	69	5.8E-12
Chinese-Belgium Gene Signature	86	5.9E-11
Asia HCC Metastases Gene Signature	98	8.2E-10
Singapore Gene Signature	15	2.2E-3
**HKU Gene Signature in Tumor Tissue**		
	**Survival** (2634 HKU Gene Signature)	
	Overlapping Genes	Enrichment p-value
Japanese Gene Signature	33	7.8E-4
Chinese-Belgium Gene Signature	90	1.3E-26
Asia HCC Metastases Gene Signature	50	2.1E-3
Singapore Gene Signature	10	6.0E-3
	**Disease Free Survival** (2169 HKU Gene Signature)	
	Overlapping Genes	Enrichment p-value
Japanese Gene Signature	35	2.7E-6
Chinese-Belgium Gene Signature	96	8.3E-38
Asia HCC Metastases Gene Signature	55	4.0E-7
Singapore Gene Signature	10	1.3E-3

‡We selected genes associated with survival or disease-free survival using nominal p-value of 0.01.

Table S2C, Using Independent HCC Studies to Validate HKU Gene Signature Identified at 0.1 Level

**HKU Gene Signature in Normal Tissue**		
	**Survival** (9775 HKU Gene Signature) ‡	
	Overlapping Genes	Enrichment p-value
Japanese Gene Signature	108	1.2E-8
Chinese-Belgium Gene Signature	157	2.8E-13
Asia HCC Metastases Gene Signature	181	8.0E-11
Singapore Gene Signature	25	8.0E-3
	**Disease Free Survival** (9912 HKU Gene Signature)	
	Overlapping Genes	Enrichment p-value
Japanese Gene Signature	112	7.5E-10
Chinese-Belgium Gene Signature	151	2.1E-10
Asia HCC Metastases Gene Signature	184	3.2E-11
Singapore Gene Signature	25	9.9E-3
**HKU Gene Signature in Tumor Tissue**		
	**Survival** (7574 HKU Gene Signature)	
	Overlapping Genes	Enrichment p-value
Japanese Gene Signature	81	2.3E-5
Chinese-Belgium Gene Signature	158	5.8E-26
Asia HCC Metastases Gene Signature	133	8.4E-6
Singapore Gene Signature	24	3.3E-4
	**Disease Free Survival** (6242 HKU Gene Signature)	
	Overlapping Genes	Enrichment p-value
Japanese Gene Signature	78	5.0E-8
Chinese-Belgium Gene Signature	158	1.7E-36
Asia HCC Metastases Gene Signature	111	4.5E-5
Singapore Gene Signature	23	3.7E-5

‡We selected genes associated with survival or disease-free survival using nominal p-value of 0.1.
