# Supplementary material for: Clinicopathologic and gene expression parameters predict liver cancer prognosis
Source: BMC Cancer. 2011 Nov 9;11:481. doi: 10.1186/1471-2407-11-481 (PMC3240666; doi:10.1186/1471-2407-11-481)
Supplement: Additional file 6 — We integrated the Hong Kong gene list with published HCC gene signatures and create the list of HCC prognosis-associated genes. The first part of the list contains genes that reached genome-wide significance level in our Hong Kong data. Herein, we did not adjust for clinicopathologic parameter in order to remain comparable to published signatures. Strict cutoff (Cox p-value ≤ 2e-6) were applied to our results, therefore, the identified genes were genome-wide significant after Bonferroni correction. The second part of the list contains genes that appeared at least twice in HCC gene lists. Here, we applied a liberal p-value cutoff (Cox p-value ≤ 0.01) to Hong Kong data. The second column of the table presents the Wald's test p-values, where "-" denotes the p-value above 0.01. The columns 3-6 are indicators for published HCC signatures, where "1" denotes present and "-" denotes absent. [file 1471-2407-11-481-S6.PDF]

Gene	HKU_Cox_pvalue	Asia	China_Belgium	Japan	Singapore
SERPINB8	0.0051	-	-	1	-
SERPINC1	0.0047	-	1	-	-
ETS2	0.0037	-	-	-	1
SERPIND1	0.00012	-	1	-	-
MSH6	0.0048	-	1	1	-
SERPINF1	0.01	-	1	-	-
RCOR1	0.0015	1	-	-	-
SERPING1	0.0047	-	1	-	-
SERPINH1	2.30E-05	-	1	-	-
IER3	1.90E-06	-	1	1	-
MSRA	0.0013	-	1	-	-
COX15	1.70E-07	-	-	-	-
ANP32B	0.00031	1	-	-	-
VCL	0.0018	-	1	-	-
PI15	2.30E-07	-	-	-	-
ACADS	0.00035	-	1	-	-
AGRN	-	1	1	-	-
CCL19	-	1	-	1	-
ITGA9	0.01	-	-	1	-
ITGB1	0.00023	1	-	-	-
GNB1	0.00031	1	-	-	-
GPLD1	1.40E-06	1	-	-	-
RALA	0.0013	-	1	-	-
ATP6V1A	0.00011	1	-	-	-
COL16A1	3.10E-05	-	-	1	-
UGT2B4	0.00056	-	1	-	-
TIMM8A	0.0062	-	-	1	-
SLC16A2	2.00E-06	-	-	-	-
AR	0.00037	-	1	1	-
RP1-21O18.1	9.80E-08	-	-	-	-
C9	0.00017	-	-	1	-
HABP2	0.0028	-	-	1	-
LYAR	0.0015	-	1	-	-
STAT3	1.40E-05	1	-	-	-
USP9X	8.40E-07	-	-	-	-
SLC6A12	0.00039	-	1	-	-
RBP5	0.0022	-	1	-	-
EHHADH	0.0041	1	1	-	-
SLC23A1	4.80E-05	1	-	-	-
SSFA2	0.0051	-	-	1	-
UGT2B10	2.00E-06	-	-	-	-
RNF10	0.0059	1	-	-	-
RNF24	1.10E-05	1	-	-	-
PINK1	0.0013	-	1	-	-
ENTPD1	0.0043	1	-	-	-
PLCB3	3.40E-05	-	-	1	-
ITIH1	0.003	-	1	-	-
CCNA2	0.01	-	1	-	-
SLC30A1	0.0025	1	-	-	-
PKLR	0.00011	-	1	1	-
CBX2	9.20E-07	-	-	-	-

CBX3	5.00E-04	-	1	-	-
ASAHL	0.0071	-	-	1	-
INPP5D	0.00096	1	-	-	-
COL4A1	6.00E-05	-	-	1	-
RDH5	0.0046	-	1	-	-
TIGD2	3.50E-05	-	-	-	1
CCT8	0.0025	-	-	1	-
CRABP2	0.00061	1	-	-	-
PLP2	0.0097	-	1	-	-
SLC2A2	0.0018	-	1	-	-
SLC19A3	3.90E-07	-	-	-	-
AKT3	1.80E-06	-	-	-	-
HYAL1	2.20E-05	-	1	-	-
ZBTB17	0.0028	-	-	1	-
PIPOX	1.30E-05	-	1	-	-
CDO1	0.00079	-	1	-	1
RACGAP1	0.00069	-	-	-	1
GRM5	0.00013	-	-	1	-
C14ORF166	9.60E-08	-	-	-	-
ARHGAP18	0.0038	-	1	-	-
PMM2	0.0097	1	-	-	-
KIF5B	0.00013	-	1	-	-
COL6A3	1.70E-05	-	-	1	-
AP2B1	0.00024	1	-	-	-
ALAS1	-	-	1	1	-
HIF1A	0.0093	-	1	-	-
AMFR	0.00049	-	1	-	-
CES2	0.0067	-	1	-	-
CES3	0.007	-	1	-	-
SLC27A2	0.0058	-	-	-	1
SLC27A5	0.0016	-	1	-	-
SLC4A4	0.0059	-	-	1	-
NCAPH	7.00E-04	-	-	1	-
NECAP2	7.10E-08	-	-	-	-
SCG5	0.00024	-	-	1	-
MECP2	2.20E-07	-	-	-	-
CD164	-	1	1	-	-
MYO3A	8.00E-07	-	-	-	-
MTHFD2	0.0012	-	1	-	-
IL1R1	0.0041	1	-	-	-
ANXA3	-	-	1	1	-
ANXA9	0.001	-	1	-	-
AKR1A1	0.00033	-	-	1	-
RGS2	0.00081	-	1	-	-
SLC34A1	0.0068	-	1	-	-
EPHA1	0.00041	1	-	-	-
AKR1D1	0.003	-	-	1	-
RAB14	0.00033	1	-	-	-
ANLN	0.0012	-	1	-	-
AGPAT3	0.0029	-	-	-	1
SDHC	1.60E-05	-	-	1	-
SDK1	1.10E-06	-	-	-	-

EPHX1	1.00E-06	-	1	-	-
IGF2R	0.0091	1	-	-	-
TWIST2	6.90E-07	-	-	-	-
DCXR	5.00E-06	-	1	-	-
SLC35D1	1.10E-05	-	1	-	-
TAF1	7.00E-04	1	-	-	-
INSIG1	-	-	1	-	1
REPS2	2.00E-07	-	-	-	-
SLIT3	0.0031	-	-	1	-
CYP2C18	1.90E-06	-	-	-	-
ALDH5A1	6.80E-07	-	-	-	-
TMEM97	5.10E-05	-	-	1	-
AMT	0.0029	-	1	-	-
AOX1	-	-	1	1	-
CNGA1	0.0012	-	-	-	1
DDR1	-	1	-	1	-
STARD10	1.10E-06	-	1	-	-
OPCML	0.0065	1	-	-	-
API5	0.0061	1	-	-	-
SRPX2	1.70E-07	-	-	-	-
SLC7A1	-	1	-	1	-
AEBP1	0.0074	-	-	1	-
AMACR	0.0038	-	1	-	-
CHORDC1	8.90E-06	1	-	-	-
C8B	-	1	-	1	-
CAT	1.10E-06	-	-	-	-
ITPR2	8.70E-06	1	1	-	-
TCF4	0.00069	-	-	1	-
PSMB9	-	1	-	1	-
AQP1	0.005	1	-	-	-
AQP9	7.70E-06	-	1	-	-
PFDN4	0.0038	-	1	-	-
SGCD	0.0028	1	-	-	-
DIAPH2	0.0022	1	-	-	-
SYTL3	1.50E-07	-	-	-	-
CRP	2.10E-05	1	-	-	-
STK39	0.0058	1	-	-	-
PSD3	1.80E-06	-	-	-	-
CENPF	0.0081	-	1	-	-
ARF4	-	1	-	1	-
SLC38A3	0.0017	-	1	-	-
KPNA1	9.10E-09	-	-	-	-
DCI	0.00054	1	-	-	-
SHC1	0.00015	1	-	-	-
GMEB1	1.10E-07	-	-	-	-
TDO2	0.0012	-	-	1	-
EPM2A	0.0017	1	-	-	-
PPP1R1A	0.0055	-	-	1	-
HDAC2	0.00038	1	1	-	-
TEAD4	0.00053	-	-	1	-
DHX15	0.0028	1	-	-	-
SC5DL	7.80E-05	-	-	1	-

WIPF1	0.0055	-	-	1	-
IQGAP1	5.00E-05	1	-	1	-
PTK7	0.0012	1	-	-	-
KIAA1333	2.00E-06	-	-	-	-
EDG4	0.0046	-	-	1	-
CFLAR	0.0019	1	-	-	-
ALDH9A1	0.00081	-	-	1	-
EMD	0.0044	-	-	1	-
SLC25A10	0.0024	-	1	-	-
MCL1	0.0024	1	-	-	-
UBE2C	3.70E-05	1	-	-	-
CLTB	0.0063	1	-	-	-
DYRK2	0.00018	-	1	-	-
CRYL1	0.00034	-	1	-	-
HIST1H4C	0.0054	-	1	-	-
PTPN18	0.0077	-	-	1	-
POLR3F	0.0063	1	-	-	-
PSPC1	0.00017	1	-	-	-
DIO1	1.60E-06	-	1	-	-
CSNK1A1	6.20E-05	1	-	-	-
GTF3C2	0.001	1	-	-	-
GHR	1.90E-05	1	-	1	-
CXCR4	0.0013	-	-	1	-
FBN1	1.60E-05	-	-	1	-
PELI1	0.0013	-	1	-	-
RNF130	0.0037	-	-	-	1
SEC14L2	5.20E-05	-	1	-	-
ACOX2	1.00E-05	-	1	-	-
LIPC	0.0044	-	-	1	-
HGF	1.30E-06	-	-	-	-
FCMD	0.00079	1	-	-	-
HPD	0.001	-	1	-	-
PAPSS1	0.0066	1	-	-	-
DKK1	0.003	1	-	-	-
NARS2	3.30E-05	-	-	1	-
RPS3	-	1	1	-	-
ICK	2.00E-05	-	-	1	-
MAPRE1	0.0069	-	1	-	-
CPB2	0.00089	-	1	-	-
IVD	3.30E-06	-	1	-	-
IYD	7.30E-07	-	-	-	-
CPT2	0.0076	-	1	-	-
PTPN2	0.0045	-	-	1	-
FAM129A	0.0013	-	-	1	-
DLGAP4	0.00081	-	-	1	-
YWHAH	0.0062	-	1	-	-
PLRG1	3.90E-07	-	-	-	-
RGS13	0.002	1	-	-	-
TRPC1	0.0059	1	-	-	-
ZNF197	0.00078	1	-	-	-
KHK	0.001	-	1	-	-
SOD2	0.0033	1	-	-	-

ANKRD46	2.70E-05	-	-	1	-
ACSL1	0.0013	1	-	-	-
MEP1B	0.0077	1	-	-	-
ACSM3	0.00022	-	-	1	-
AS3MT	0.00018	-	-	-	1
CYP4F11	4.00E-04	-	1	-	-
CRY2	0.00014	1	-	-	-
FGL1	2.10E-08	-	-	-	-
DNAJC10	0.002	-	-	-	1
LPP	0.00086	-	-	1	-
CSDA	1.90E-05	-	1	-	-
CUTL2	-	-	1	1	-
TM7SF2	5.30E-06	-	-	1	-
ACTR3	0.00075	1	1	-	-
GPR161	3.70E-07	-	-	-	-
PCYT2	-	-	1	1	-
KCNJ3	0.0096	-	-	1	-
KCNJ8	0.0012	1	-	-	-
WARS	1.30E-05	1	-	-	-
ELF3	0.00034	1	-	-	-
DENND3	1.90E-06	-	-	-	-
ELL2	0.00039	1	-	-	1
LGALS8	0.00082	1	-	-	-
LAMB1	0.00065	-	1	-	-
HAAO	0.0027	-	-	1	-
HAGH	0.00081	-	1	-	-
ZNF410	0.00045	1	-	-	-
LAMP2	0.0057	1	-	-	-
HAO1	0.00059	-	1	-	-
DPYS	3.00E-05	-	1	-	-
C4BPB	-	-	1	1	-
MTSS1	-	1	1	-	-
EMP2	0.00021	-	-	1	-
NTS	-	-	1	1	-
RBM34	0.0051	-	-	1	-
DNAJC7	0.0043	1	-	-	-
SPACA3	0.0074	-	-	-	1
SGMS1	1.70E-06	-	-	-	-
CALD1	0.0013	1	-	-	-
OTC	0.00024	-	1	-	-
SSR1	0.0072	1	-	-	-
HMGB2	1.50E-07	-	1	-	-
HMGCL	0.00013	-	-	1	-
HMGCR	-	1	-	1	-
GGCX	0.0011	-	-	1	-
GTSE1	2.00E-06	-	-	-	-
TCAG7.1188	3.20E-07	-	-	-	-
BRP44	2.20E-05	-	-	1	-
WDR23	1.70E-05	-	-	1	-
WDR26	6.90E-07	-	-	-	-
WDR32	4.40E-07	-	-	-	-
PLG	0.0026	-	-	1	-

TPM4	0.0026	1	-	-	-
DSG1	0.00041	-	1	-	-
LEPREL2	4.90E-07	-	-	-	-
ARHGDIA	0.0062	1	-	-	-
SMARCA5	0.0043	1	-	-	-
PBX1	-	1	1	-	-
FOSL2	1.60E-06	-	-	-	-
PCCB	0.00022	-	1	-	-
ABI2	4.50E-08	-	-	-	-
PCK1	-	-	1	1	-
PCK2	9.20E-07	-	-	-	-
CYP3A4	0.0055	-	1	-	-
CDC40	0.006	1	-	-	-
THBS1	0.0027	1	-	-	-
SELENBP1	3.30E-05	-	1	1	-
LSM8	0.00039	-	1	-	-
SGPL1	0.00017	-	1	-	-
ATP11C	8.00E-07	-	-	-	-
HES4	1.70E-05	-	1	-	-
RGN	0.0018	-	1	-	-
PDK1	0.0019	1	-	-	-
FMO3	0.00025	-	1	-	-
RLF	0.00053	-	-	1	-
SLCO3A1	0.00045	1	-	-	-
HSPA5	3.30E-05	1	-	-	-
C1ORF9	0.00019	1	-	-	-
F13B	0.0018	-	1	-	-
GJB1	7.60E-05	-	1	1	-
S100A6	0.0052	-	1	-	-
LARP6	0.0098	-	-	-	1
CTNND2	0.0076	-	-	1	-
ADD3	0.0017	-	-	1	-
ADH5	2.00E-04	-	-	1	-
ADH6	3.00E-05	-	-	1	-
PER2	2.00E-04	1	-	-	-
ATP2C1	0.0042	-	-	1	-
NDRG2	0.0039	-	1	-	-
HPCAL1	2.00E-06	-	-	-	-
LOXL2	3.90E-05	-	-	1	-
SMURF2	0.0062	-	-	-	1
RASL12	1.20E-06	-	-	-	-
TES	2.30E-05	-	1	-	-
PGM2L1	0.00068	1	-	-	-
PPM1D	0.0025	1	-	-	-
PPM1G	0.0037	1	-	-	-
GLYAT	1.10E-06	1	1	-	-
SUCLG1	0.0039	-	-	1	-
TTK	0.00069	1	1	-	-
SP100	0.0045	-	-	1	-
CHSY1	0.00018	-	-	1	-
ABCG8	0.01	-	1	-	-
UNC5A	3.80E-07	-	-	-	-

LMBRD2	0.00018	-	-	-	1
CDC42SE1	4.00E-05	-	-	-	1
DUSP4	2.50E-09	-	-	-	-
DUSP5	6.80E-07	-	-	1	-
SLC20A2	0.00049	1	-	-	-
