# Supplementary material for: Clinicopathologic and gene expression parameters predict liver cancer prognosis
Source: BMC Cancer. 2011 Nov 9;11:481. doi: 10.1186/1471-2407-11-481 (PMC3240666; doi:10.1186/1471-2407-11-481)
Supplement: Additional file 7 — Supplementary Table 3B [file 1471-2407-11-481-S7.PDF]

Gene	HKU_Cox_pvalue	Asia	China_Belgium	Japan	Singapore
SERPINC1	0.0022	-	1	-	-
ETS2	0.0023	-	-	-	1
SERPIND1	4.00E-04	-	1	-	-
MSH6	0.0037	-	1	1	-
SERPINH1	0.0012	-	1	-	-
IER3	2.40E-05	-	1	1	-
MSRA	0.00085	-	1	-	-
COX15	6.10E-07	-	-	-	-
ANP32B	6.90E-05	1	-	-	-
VCL	0.0026	-	1	-	-
ACADS	0.0036	-	1	-	-
AGRN	-	1	1	-	-
CCL19	-	1	-	1	-
AGXT2L1	1.30E-06	-	-	-	-
ATP5C1	0.0074	-	1	-	-
NTRK3	5.00E-07	-	-	-	-
ITGA9	0.0033	-	-	1	-
ITGB1	0.00032	1	-	-	-
GNB1	0.00011	1	-	-	-
GPLD1	2.90E-06	1	-	-	-
ATP6V1A	1.80E-05	1	-	-	-
COL16A1	2.10E-05	-	-	1	-
UGT2B4	0.0012	-	1	-	-
TIMM8A	0.0021	-	-	1	-
AR	0.0045	-	1	1	-
RP1-21O18.1	5.70E-07	-	-	-	-
C9	0.00011	-	-	1	-
USP14	0.0071	-	-	1	-
HABP2	0.001	-	-	1	-
LYAR	0.0029	-	1	-	-
AKAP12	9.80E-07	-	-	-	-
STAT3	1.20E-05	1	-	-	-
USP9X	1.50E-06	-	-	-	-
SLC6A12	0.0013	-	1	-	-
RBM6	0.006	1	-	-	-
RBP5	0.003	-	1	-	-
EHHADH	0.0034	1	1	-	-
SLC23A1	3.80E-05	1	-	-	-
AIM1	0.0021	1	-	-	-
RNF10	0.0038	1	-	-	-
RNF24	0.00054	1	-	-	-
PINK1	0.0015	-	1	-	-
ENTPD1	0.0012	1	-	-	-
PLCB3	4.10E-05	-	-	1	-
ITIH1	0.0085	-	1	-	-
SLC30A1	0.0011	1	-	-	-
PKLR	3.00E-04	-	1	1	-
CBX3	5.30E-05	-	1	-	-
INPP5D	5.20E-06	1	-	-	-
CCNT1	0.0085	1	-	-	-
COL4A1	0.0018	-	-	1	-

SMAD2	0.004	1	-	-	-
RDH5	0.0056	-	1	-	-
TIGD2	0.00015	-	-	-	1
TGFBR1	1.40E-06	-	-	-	-
CCT5	0.0052	-	1	-	-
CRABP2	0.00072	1	-	-	-
SLC2A2	0.0021	-	1	-	-
ARRDC2	6.00E-07	-	-	-	-
SLC19A3	1.90E-07	-	-	-	-
PRKCE	0.0044	1	-	-	-
HYAL1	0.00011	-	1	-	-
ZBTB17	0.0017	-	-	1	-
PIPOX	2.20E-05	-	1	-	-
CDO1	0.0012	-	1	-	1
RACGAP1	3.30E-05	-	-	-	1
GRM5	0.00019	-	-	1	-
C14ORF166	2.10E-07	-	-	-	-
ARHGAP18	0.0043	-	1	-	-
PMM2	0.0043	1	-	-	-
KIF5B	4.00E-04	-	1	-	-
APPBP2	1.40E-06	-	-	-	-
COL6A3	5.10E-05	-	-	1	-
AP2B1	0.00017	1	-	-	-
ALAS1	-	-	1	1	-
AMFR	0.0011	-	1	-	-
SLC27A2	0.0089	-	-	-	1
SLC4A4	0.0049	-	-	1	-
NCAPH	7.00E-04	-	-	1	-
NECAP2	1.70E-06	-	-	-	-
SCG5	0.00029	-	-	1	-
MECP2	9.20E-08	-	-	-	-
CD164	-	1	1	-	-
MTHFD2	0.0055	-	1	-	-
ANXA3	-	-	1	1	-
ANXA9	0.0025	-	1	-	-
AKR1A1	0.00024	-	-	1	-
RGS2	0.00079	-	1	-	-
SLC34A1	0.0081	-	1	-	-
EPHA1	0.00087	1	-	-	-
RAB14	2.50E-05	1	-	-	-
RAB28	0.0054	1	-	-	-
ANLN	0.00024	-	1	-	-
MASP2	0.0081	-	1	-	-
SDHC	4.10E-05	-	-	1	-
EPHX1	1.60E-06	-	1	-	-
DCXR	4.80E-06	-	1	-	-
PCMT1	0.0051	1	-	-	-
SLC35D1	1.40E-05	-	1	-	-
TAF1	0.00056	1	-	-	-
INSIG1	-	-	1	-	1
REPS2	3.40E-07	-	-	-	-
SLIT3	0.0017	-	-	1	-

MFAP3	0.0038	1	-	-	-
ALDH5A1	1.50E-06	-	-	-	-
TMEM97	5.60E-05	-	-	1	-
SFRS11	0.0018	1	-	-	-
AOX1	-	-	1	1	-
CHD4	0.0019	1	-	-	-
CHD9	0.0026	1	-	-	-
CNGA1	0.0018	-	-	-	1
DDR1	-	1	-	1	-
STARD10	3.70E-06	-	1	-	-
OPCML	0.0075	1	-	-	-
SLC7A1	-	1	-	1	-
AEBP1	0.0056	-	-	1	-
CHORDC1	0.0039	1	-	-	-
C8B	-	1	-	1	-
ITPR2	9.90E-06	1	1	-	-
MEGF9	1.70E-06	-	-	-	-
TCF4	0.00026	-	-	1	-
PSMB9	-	1	-	1	-
AQP1	0.0043	1	-	-	-
AQP9	1.90E-05	-	1	-	-
PFDN4	0.00099	-	1	-	-
SGCD	0.0012	1	-	-	-
DIAPH2	0.002	1	-	-	-
SYTL3	1.10E-09	-	-	-	-
SPATS2	6.40E-07	-	-	-	-
CRP	0.00011	1	-	-	-
STK39	0.0082	1	-	-	-
CUL4B	0.0067	-	-	-	1
ARF4	-	1	-	1	-
SLC38A3	0.0082	-	1	-	-
KPNA1	6.10E-08	-	-	-	-
DCI	7.90E-05	1	-	-	-
PLEKHO1	1.80E-06	-	-	-	-
SHC1	0.0015	1	-	-	-
GMEB1	1.00E-07	-	-	-	-
TDO2	0.003	-	-	1	-
EPM2A	0.0064	1	-	-	-
DST	0.0046	-	-	1	-
HDAC2	0.00034	1	1	-	-
TEAD4	0.001	-	-	1	-
SC5DL	0.00032	-	-	1	-
WIPF1	0.0069	-	-	1	-
SLC39A8	6.70E-07	-	-	-	-
IQGAP1	4.10E-05	1	-	1	-
PTK7	0.0012	1	-	-	-
EDG4	0.00048	-	-	1	-
CFLAR	0.00021	1	-	-	-
ALDH9A1	0.0029	-	-	1	-
EMD	9.20E-05	-	-	1	-
NDUFS2	0.0051	-	1	-	-
MCL1	0.00038	1	-	-	-

UBE2C	3.10E-05	1	-	-	-
CLTB	9.60E-05	1	-	-	-
DYRK2	0.00014	-	1	-	-
FER	0.0061	1	-	-	-
CRYL1	4.00E-04	-	1	-	-
HIST1H4C	0.0017	-	1	-	-
POLR3F	0.0071	1	-	-	-
PSPC1	0.00034	1	-	-	-
DIO1	3.40E-06	-	1	-	-
CSNK1A1	2.40E-06	1	-	-	-
GTF3C2	0.0021	1	-	-	-
CSNK1G3	0.01	-	-	-	1
GHR	6.70E-05	1	-	1	-
CXCR4	0.001	-	-	1	-
FBN1	0.00011	-	-	1	-
PELI1	0.0032	-	1	-	-
RNF130	0.0062	-	-	-	1
SEC14L2	8.00E-04	-	1	-	-
ACOX2	2.90E-05	-	1	-	-
LIPC	0.0023	-	-	1	-
KIAA1600	1.60E-06	-	-	-	-
HPD	0.00035	-	1	-	-
PAPSS1	0.0028	1	-	-	-
DKK1	0.01	1	-	-	-
NARS2	3.10E-05	-	-	1	-
MAP4K1	0.0028	-	1	-	-
RPS3	-	1	1	-	-
ICK	5.20E-05	-	-	1	-
SPTLC2	7.40E-07	-	-	-	-
POU2F2	0.0085	1	-	-	-
DLG7	0.0053	-	1	-	-
CPB2	0.00028	-	1	-	-
IVD	4.10E-05	-	1	-	-
MLSTD2	8.00E-07	-	-	-	-
PABPC1	0.01	1	-	-	-
C4ORF19	1.20E-07	-	-	-	-
CNOT4	0.0061	1	-	-	-
NDE1	1.80E-06	-	-	-	-
PTPN2	0.005	-	-	1	-
FAM129A	0.0064	-	-	1	-
DLGAP4	0.00072	-	-	1	-
BUB3	0.005	-	1	-	-
PLRG1	8.80E-08	-	-	-	-
RGS13	8.10E-05	1	-	-	-
TRPC1	0.0047	1	-	-	-
ZNF197	0.00012	1	-	-	-
KHK	0.0089	-	1	-	-
FAM137B	6.80E-07	-	-	-	-
ANKRD46	3.20E-05	-	-	1	-
ACSL1	0.00026	1	-	-	-
MEP1B	0.0088	1	-	-	-
ACSM3	2.00E-04	-	-	1	-

AS3MT	0.0012	-	-	-	1
NEK2	0.0025	1	-	-	-
CYP4F11	9.50E-05	-	1	-	-
SOX4	1.90E-06	-	-	-	-
LEP	0.0032	1	-	-	-
CRY2	0.0035	1	-	-	-
FGL1	8.00E-08	-	-	-	-
JUNB	1.40E-06	-	-	-	-
DNAJC10	0.0034	-	-	-	1
LPP	0.00052	-	-	1	-
CSDA	0.00037	-	1	-	-
CUTL2	-	-	1	1	-
TM7SF2	9.70E-05	-	-	1	-
ACTR3	6.00E-04	1	1	-	-
PCYT2	-	-	1	1	-
KCNJ8	0.0011	1	-	-	-
WARS	9.60E-06	1	-	-	-
ELF3	0.00036	1	-	-	-
DENND3	1.80E-07	-	-	-	-
ELL2	0.00022	1	-	-	1
LGALS8	0.001	1	-	-	-
LAMB1	0.001	-	1	-	-
HAAO	0.0068	-	-	1	-
HAGH	0.0029	-	1	-	-
ZNF410	0.00021	1	-	-	-
LAMP2	0.0063	1	-	-	-
HAO1	0.0057	-	1	-	-
DPYS	9.40E-05	-	1	-	-
BRMS1	1.10E-06	-	-	-	-
NF1	0.0092	-	1	-	-
NHS	1.80E-06	-	-	-	-
KSR1	6.00E-07	-	-	-	-
TNK2	0.0095	-	-	1	-
C4BPB	-	-	1	1	-
MTSS1	-	1	1	-	-
EMP2	3.30E-05	-	-	1	-
NTS	-	-	1	1	-
HNRPH1	0.0068	1	-	-	-
RBM34	0.0022	-	-	1	-
CREB1	0.0013	-	-	1	-
DNAJC7	2.00E-04	1	-	-	-
SPACA3	0.0093	-	-	-	1
SGMS1	6.20E-07	-	-	-	-
CALD1	0.00014	1	-	-	-
OTC	8.10E-05	-	1	-	-
SSR1	0.0025	1	-	-	-
HMGB2	3.00E-08	-	1	-	-
HMGCL	0.0017	-	-	1	-
HMGCR	0.0092	1	-	1	-
GGCX	0.00041	-	-	1	-
POLA1	6.60E-07	-	-	-	-
BRP44	3.40E-05	-	-	1	-

WDR23	3.00E-04	-	-	1	-
WDR32	3.40E-07	-	-	-	-
DSC2	0.0015	1	-	-	-
PLG	0.0016	-	-	1	-
DSG1	0.00064	-	1	-	-
LEPREL2	1.60E-06	-	-	-	-
ARHGDIA	0.0066	1	-	-	-
PBX1	-	1	1	-	-
FOSL2	5.30E-07	-	-	-	-
STX6	2.00E-06	-	-	-	-
STYX	1.90E-06	-	-	-	-
PCCB	0.00012	-	1	-	-
DERL3	0.0032	1	-	-	-
ABI2	8.90E-10	-	-	-	-
PCK1	-	-	1	1	-
CDC40	0.0022	1	-	-	-
THBS1	0.0065	1	-	-	-
RDH14	0.0064	1	-	-	-
SELENBP1	7.40E-05	-	1	1	-
LSM8	2.30E-05	-	1	-	-
SGPL1	0.00015	-	1	-	-
HES4	3.00E-05	-	1	-	-
RGN	0.0067	-	1	-	-
UNKL	0.0029	-	1	-	-
PDK1	0.00059	1	-	-	-
FMO3	0.00033	-	1	-	-
RLF	0.00022	-	-	1	-
SLCO3A1	0.0027	1	-	-	-
HSPA5	1.10E-05	1	-	-	-
C1ORF9	9.10E-05	1	-	-	-
DDX24	1.20E-06	-	-	-	-
DDX3Y	0.0049	1	-	-	-
F13B	0.0046	-	1	-	-
GJB1	0.00063	-	1	1	-
S100A6	0.0063	-	1	-	-
LARP4	1.10E-06	-	-	-	-
ADD3	0.00044	-	-	1	-
SET	0.0049	-	1	-	-
ADH5	0.00018	-	-	1	-
ADH6	0.00012	-	-	1	-
PER2	0.00045	1	-	-	-
ATP2C1	0.0052	-	-	1	-
HPCAL1	5.90E-07	-	-	-	-
LOXL2	0.00084	-	-	1	-
RASL12	1.70E-06	-	-	-	-
TES	5.30E-05	-	1	-	-
PGM2L1	0.0038	1	-	-	-
ABCB10	9.70E-07	-	-	-	-
PPM1D	0.00013	1	-	-	-
PPM1G	0.0011	1	-	-	-
VAMP3	0.0088	1	-	-	-
GLYAT	5.50E-06	1	1	-	-

SUCLG1	0.00048	-	-	1	-
TTK	1.70E-05	1	1	-	-
SP100	0.00057	-	-	1	-
CHSY1	0.0023	-	-	1	-
UNC5A	1.10E-06	-	-	-	-
LMBRD2	6.10E-05	-	-	-	1
CDC42SE1	7.80E-05	-	-	-	1
DUSP4	9.80E-08	-	-	-	-
DUSP5	6.90E-07	-	-	1	-
SLC20A2	0.0032	1	-	-	-
