# Supplementary material for: Clinicopathologic and gene expression parameters predict liver cancer prognosis
Source: BMC Cancer. 2011 Nov 9;11:481. doi: 10.1186/1471-2407-11-481 (PMC3240666; doi:10.1186/1471-2407-11-481)
Supplement: Additional file 8 — Supplementary Table 3C [file 1471-2407-11-481-S8.PDF]

Gene	HKU_Cox_pvalue	Asia	China_Belgium	Japan	Singapore
SERPINA3	0.00042	1	-	-	-
SERPINC1	0.0027	-	1	-	-
MSH6	-	-	1	1	-
NONO	2.10E-08	-	-	-	-
RCOR1	0.0044	1	-	-	-
SERPINH1	0.0045	-	1	-	-
IER3	8.20E-05	-	1	1	-
ANP32B	3.70E-06	1	-	-	-
FOXQ1	4.80E-07	-	-	-	-
VCL	6.10E-05	-	1	-	-
CCKBR	1.50E-07	-	-	-	-
BCL2	0.0091	-	-	1	-
AGRN	0.0077	1	1	-	-
CCL19	0.0098	1	-	1	-
ITGA2	2.10E-07	-	-	-	-
ITGB1	0.0039	1	-	-	-
GPLD1	0.00055	1	-	-	-
RALA	0.0034	-	1	-	-
PIGK	0.0035	-	-	1	-
PIM1	6.30E-09	1	-	-	-
SLC16A1	0.0089	1	-	-	-
BCORL1	1.30E-07	-	-	-	-
AR	-	-	1	1	-
C3	0.00075	1	-	-	-
C6	6.80E-05	1	-	-	-
C9	0.0046	-	-	1	-
F9	0.0011	-	-	1	-
STAT3	0.0096	1	-	-	-
SLC6A19	1.40E-07	-	-	-	-
EHHADH	1.10E-05	1	1	-	-
NAP1L1	0.001	-	1	-	-
RNF24	0.0035	1	-	-	-
PINK1	0.0023	-	1	-	-
PLCB3	4.20E-05	-	-	1	-
ITIH1	0.0016	-	1	-	-
CCNA2	0.009	-	1	-	-
CLIC1	0.00013	-	1	-	-
PKLR	-	-	1	1	-
CBX3	0.0052	-	1	-	-
CCT8	0.0027	-	-	1	-
BACH1	1.50E-06	-	-	-	-
CRABP2	0.0059	1	-	-	-
SLC2A2	0.00051	-	1	-	-
SULT2A1	0.006	-	1	-	-
PIPOX	0.00053	-	1	-	-
CDK4	0.00089	-	1	-	-
TEX10	9.00E-11	-	-	-	-
CDO1	0.00057	-	1	-	1
RACGAP1	0.0098	-	-	-	1
C20ORF70	1.40E-06	-	-	-	-
APOA5	0.00097	-	1	-	-

TXNRD1	0.0038	1	-	-	-
AP2B1	7.00E-04	1	-	-	-
ALAS1	0.0041	-	1	1	-
AMFR	0.00089	-	1	-	-
CES2	0.00081	-	1	-	-
CES3	0.01	-	1	-	-
SLC27A2	0.00036	-	-	-	1
SLC27A5	1.30E-06	-	1	-	-
RWDD1	0.0043	-	1	-	-
ZER1	0.00055	-	-	1	-
CD164	-	1	1	-	-
ANXA3	-	-	1	1	-
EPHA4	0.0038	-	-	1	-
RAB14	0.0037	1	-	-	-
ANLN	0.0059	-	1	-	-
MASP2	0.00022	-	1	-	-
PPP3CC	4.30E-07	-	-	-	-
CD2AP	0.00059	-	1	-	-
DCXR	0.0015	-	1	-	-
SLC35D1	0.0024	-	1	-	-
AGL	0.0061	-	1	-	-
TAF1	0.00037	1	-	-	-
INSIG1	0.0035	-	1	-	1
AOX1	-	-	1	1	-
CHD4	0.00067	1	-	-	-
EEF1E1	0.00021	-	1	-	-
ACIN1	3.10E-05	1	-	-	-
CNGA1	0.00075	-	-	-	1
DDR1	-	1	-	1	-
HNRPK	0.0099	1	-	-	-
BLMH	2.00E-06	-	-	-	-
SLC7A1	-	1	-	1	-
MARCKS	2.10E-07	-	1	-	-
C8B	7.00E-04	1	-	1	-
SLC37A4	0.0054	-	-	1	-
ITPR2	-	1	1	-	-
DDHD2	0.009	1	-	-	-
FNIP1	2.30E-07	-	-	-	-
PSMB9	-	1	-	1	-
PRR3	5.60E-08	-	-	-	-
PSMD5	2.30E-07	-	-	-	-
DIAPH2	0.0015	1	-	-	-
SPATS2	2.60E-08	-	-	-	-
IPMK	4.80E-07	-	-	-	-
CUL4B	0.00048	-	-	-	1
ARF4	-	1	-	1	-
SHC1	3.70E-05	1	-	-	-
ZFAND3	2.00E-06	-	-	-	-
TDO2	0.00087	-	-	1	-
HDAC2	-	1	1	-	-
TEAD1	0.0021	1	-	-	-
TEAD4	0.0035	-	-	1	-

SC5DL	0.0014	-	-	1	-
CKS2	0.0067	-	1	-	-
IQGAP1	-	1	-	1	-
PTK7	8.00E-04	1	-	-	-
EMD	0.0033	-	-	1	-
CABC1	0.0068	-	1	-	-
MID1IP1	1.40E-11	-	-	-	-
F12	0.00011	-	1	-	-
UBE2C	0.00065	1	-	-	-
DYRK2	0.0053	-	1	-	-
HIST1H4C	2.40E-05	-	1	-	-
CHERP	6.30E-05	-	-	1	-
GTF3C2	0.0044	1	-	-	-
CSNK1G3	5.40E-05	-	-	-	1
RAI14	6.60E-07	-	-	-	-
GHR	0.0024	1	-	1	-
C20ORF166	0.0013	-	1	-	-
ROD1	0.0042	-	1	-	-
PCTK1	5.90E-07	-	-	-	-
ACOX2	1.10E-06	-	1	-	-
HGD	0.00038	-	1	-	-
HPD	0.0011	-	1	-	-
RPS3	0.0046	1	1	-	-
RPS9	0.0091	-	1	-	-
RPL17	8.20E-05	-	1	-	-
RPL27	0.005	-	1	-	-
RPL35	3.20E-06	-	1	-	-
RPL38	0.00099	-	1	-	-
CYB5A	0.0019	-	-	1	-
POU2F1	4.10E-07	-	-	-	-
DUSP13	7.50E-07	-	-	-	-
MAPRE1	0.0038	-	1	-	-
CPA1	0.00058	1	-	-	-
DLG7	0.00036	-	1	-	-
IVD	0.00019	-	1	-	-
PABPC1	0.00092	1	-	-	-
CPT2	1.60E-05	-	1	-	-
MAGEA10	1.40E-06	-	-	-	-
JARID2	9.10E-07	-	-	-	-
DLGAP4	0.00016	-	-	1	-
FRAT2	0.01	1	-	-	-
RGS22	4.80E-07	-	-	-	-
UGDH	0.0014	1	-	-	-
ACSL1	0.00056	1	-	-	-
TSC22D2	0.0098	-	-	1	-
OASL	0.0075	-	-	-	1
KHDRBS1	0.0027	-	1	-	-
CRY2	0.0049	1	-	-	-
ZNF24	0.0014	1	-	-	-
LPP	0.00083	-	-	1	-
CSDA	0.0095	-	1	-	-
CUTL2	0.0017	-	1	1	-

TM7SF2	0.0013	-	-	1	-
ACTR2	0.0052	-	-	1	-
ACTR3	-	1	1	-	-
PCYT2	-	-	1	1	-
MMD	0.00072	-	1	-	-
ELL2	-	1	-	-	1
LGALS8	0.00058	1	-	-	-
B3GNT8	3.20E-07	-	-	-	-
HCG_1983332	7.40E-09	-	-	-	-
DPYS	0.0011	-	1	-	-
EML4	1.00E-06	-	-	-	-
C4BPB	-	-	1	1	-
MTSS1	-	1	1	-	-
NTS	-	-	1	1	-
SPACA3	0.0074	-	-	-	1
CCDC6	0.00011	-	-	1	-
OTC	6.20E-06	-	1	-	-
SSR1	0.0026	1	-	-	-
HMGB2	0.0066	-	1	-	-
HMGCR	-	1	-	1	-
GGCX	0.0095	-	-	1	-
MMP3	4.70E-08	-	-	-	-
PAH	0.0056	-	1	-	-
RPS18	0.00037	-	1	-	-
RPS3A	0.00018	-	1	-	-
WDR23	0.0023	-	-	1	-
DSC2	0.0015	1	-	-	-
PLG	7.00E-04	-	-	1	-
TPM4	0.0085	1	-	-	-
SMARCA5	0.0056	1	-	-	-
SMARCC1	8.90E-08	-	-	-	-
PBX1	-	1	1	-	-
C21ORF56	7.90E-07	-	-	-	-
RXRG	0.002	1	-	-	-
PCCB	0.0061	-	1	-	-
PCK1	-	-	1	1	-
SELENBP1	0.0062	-	1	1	-
FMO3	0.0033	-	1	-	-
RLF	0.0029	-	-	1	-
RP11-167P23.2	1.60E-07	-	-	-	-
GJB1	-	-	1	1	-
SET	0.0029	-	1	-	-
ASXL2	5.90E-07	-	-	-	-
PES1	0.0043	1	-	-	-
NDRG2	0.00011	-	1	-	-
TES	0.0013	-	1	-	-
PGM2L1	0.0085	1	-	-	-
GLYAT	0.00013	1	1	-	-
SUCLG1	0.00054	-	-	1	-
ABCA6	0.0078	-	1	-	-
TTK	-	1	1	-	-
ABCC2	0.0042	-	1	-	-

ABCF2	0.0044	1	-	-	-
LMBRD2	0.0024	-	-	-	1
SLC20A2	0.0016	1	-	-	-
HCG_1992539	4.20E-07	-	-	-	-
