# Supplementary material for: Clinicopathologic and gene expression parameters predict liver cancer prognosis
Source: BMC Cancer. 2011 Nov 9;11:481. doi: 10.1186/1471-2407-11-481 (PMC3240666; doi:10.1186/1471-2407-11-481)
Supplement: Additional file 9 — Supplementary Table 3D [file 1471-2407-11-481-S9.PDF]

Gene	HKU_Cox_pvalue	Asia	China_Belgium	Japan	Singapore
SERPINA3	1.80E-05	1	-	-	-
CCDC109A	1.40E-06	-	-	-	-
NOL7	0.0012	-	-	1	-
SERPINE2	1.40E-06	-	-	-	-
MSH6	-	-	1	1	-
SERPINF1	0.0065	-	1	-	-
SERPING1	0.00017	-	1	-	-
SERPINH1	0.0011	-	1	-	-
IER3	0.0011	-	1	1	-
MSRA	0.0053	-	1	-	-
ANP32B	6.90E-05	1	-	-	-
VCL	0.0051	-	1	-	-
BCL2	0.0046	-	-	1	-
MTA3	2.00E-06	-	-	-	-
AGRN	-	1	1	-	-
CCL19	-	1	-	1	-
GPLD1	0.00018	1	-	-	-
GNG4	5.20E-07	-	-	-	-
RALA	0.0024	-	1	-	-
FUBP1	0.0098	1	-	-	-
PIGK	0.0043	-	-	1	-
PIM1	2.10E-07	1	-	-	-
SLC16A1	0.004	1	-	-	-
IGF1	0.00013	-	-	1	-
AR	-	-	1	1	-
C3	7.10E-05	1	-	-	-
C5	0.005	-	-	1	-
C6	1.90E-06	1	-	-	-
C9	0.00057	-	-	1	-
USP14	0.00071	-	-	1	-
F9	2.50E-07	-	-	1	-
HABP2	0.01	-	-	1	-
XDH	1.70E-06	-	-	-	-
EHHADH	0.00028	1	1	-	-
NAP1L1	0.00044	-	1	-	-
PINK1	0.00029	-	1	-	-
PLCB1	2.60E-09	-	-	-	-
PLCB3	0.0025	-	-	1	-
ITIH1	0.007	-	1	-	-
CLIC1	2.10E-05	-	1	-	-
PKLR	-	-	1	1	-
HERC4	7.60E-08	-	-	-	-
SLC1A7	1.70E-06	-	-	-	-
CCNT1	5.20E-05	1	-	-	-
SMAD2	0.0037	1	-	-	-
TIGD2	7.00E-04	-	-	-	1
GRIN2D	0.0039	1	-	-	-
NOL5A	0.0081	-	1	-	-
CCT3	0.0015	1	-	-	-
CCT8	0.00095	-	-	1	-
BACH1	1.00E-06	-	-	-	-

CRABP2	7.60E-05	1	-	-	-
SAA4	1.20E-06	-	-	-	-
SLC2A2	0.00024	-	1	-	-
NOLC1	7.40E-05	1	-	-	-
SULT2A1	0.0042	-	1	-	-
PRKCE	0.0091	1	-	-	-
HYAL1	0.002	-	1	-	-
PIPOX	0.00041	-	1	-	-
ZBTB34	1.80E-06	-	-	-	-
CDK4	6.00E-04	-	1	-	-
CDK9	0.0034	1	-	-	-
TEX10	6.20E-07	-	-	-	-
CDO1	0.0069	-	1	-	1
RACGAP1	0.005	-	-	-	1
GRM5	0.0011	-	-	1	-
APOC3	0.0053	-	1	-	-
TXNRD1	0.0099	1	-	-	-
AP2B1	8.20E-07	1	-	-	-
ALAS1	0.0055	-	1	1	-
CES2	0.0052	-	1	-	-
CES3	0.0092	-	1	-	-
HRASLS3	0.0012	-	-	1	-
SLC27A2	0.0015	-	-	-	1
SLC27A5	1.10E-05	-	1	-	-
CD164	-	1	1	-	-
MTHFD2	0.0027	-	1	-	-
ANXA3	-	-	1	1	-
EPHA1	0.0025	1	-	-	-
AKR1D1	0.0026	-	-	1	-
RAB14	0.0013	1	-	-	-
ANLN	0.0055	-	1	-	-
MASP2	0.00023	-	1	-	-
RHBG	0.0011	-	1	-	-
EPHX1	0.0032	-	1	-	-
DCXR	0.0031	-	1	-	-
SLC35D1	0.00039	-	1	-	-
AGL	0.0021	-	1	-	-
INSIG1	0.00056	-	1	-	1
BTN2A1	6.60E-07	-	-	-	-
AOX1	-	-	1	1	-
EEF1E1	0.00022	-	1	-	-
ACIN1	0.00036	1	-	-	-
CNGA1	0.0043	-	-	-	1
DDR1	-	1	-	1	-
APCS	0.0099	-	1	-	-
API5	0.0072	1	-	-	-
CEP192	1.00E-06	-	-	-	-
LCOR	4.40E-07	-	-	-	-
SLC7A1	-	1	-	1	-
MARCKS	3.70E-06	-	1	-	-
CACNB4	9.20E-08	-	-	-	-
C8B	0.0023	1	-	1	-

PRCP	0.0036	1	-	-	-
ITPR2	-	1	1	-	-
BCAT1	0.0067	-	1	-	-
PSMB9	-	1	-	1	-
PRR3	8.70E-07	-	-	-	-
PSMD5	1.60E-06	-	-	-	-
DIAPH2	3.00E-04	1	-	-	-
MAFB	0.0046	-	-	-	1
CUL4B	0.00093	-	-	-	1
ARF4	-	1	-	1	-
DEK	0.0029	-	1	-	-
SHC1	0.00011	1	-	-	-
ALDH8A1	1.10E-06	-	-	-	-
TDO2	0.00088	-	-	1	-
HDAC2	-	1	1	-	-
SC5DL	0.0017	-	-	1	-
DEFA6	0.0033	1	-	-	-
NDUFA5	0.0014	1	-	-	-
IQGAP1	-	1	-	1	-
PTK7	0.002	1	-	-	-
MID1IP1	7.50E-07	-	-	-	-
F12	1.50E-05	-	1	-	-
CAPZA1	0.0065	1	-	-	-
UBE2C	0.0078	1	-	-	-
DYRK2	0.01	-	1	-	-
HIST1H4C	7.00E-04	-	1	-	-
GTF3C2	0.0082	1	-	-	-
GHR	7.70E-05	1	-	1	-
RNF130	7.10E-05	-	-	-	1
ROD1	0.00014	-	1	-	-
CSNK2A1	1.50E-06	-	-	-	-
SEC14L2	0.0039	-	1	-	-
ACOX2	2.30E-06	-	1	-	-
HGD	0.0062	-	1	-	-
FCMD	0.0075	1	-	-	-
HPD	0.00088	-	1	-	-
DKK1	0.00039	1	-	-	-
RPS3	0.00045	1	1	-	-
RPS6	0.0056	-	1	-	-
RPS7	0.0061	-	1	-	-
RPS9	0.0081	-	1	-	-
RPL31	0.00071	-	1	-	-
RPL35	1.30E-07	-	1	-	-
CYB5A	0.0014	-	-	1	-
SMC3	1.10E-07	-	-	-	-
MAPRE1	0.0017	-	1	-	-
DLG7	0.0027	-	1	-	-
IVD	1.10E-05	-	1	-	-
CPN2	0.00041	-	1	-	-
C1ORF182	1.90E-06	-	-	-	-
PABPC1	0.0032	1	-	-	-
CPT2	0.00013	-	1	-	-

PLXDC1	2.00E-04	1	-	-	-
PTPN1	0.0092	1	-	-	-
FRAT2	0.0012	1	-	-	-
KHK	0.001	-	1	-	-
ACSL1	0.00017	1	-	-	-
KHDRBS1	0.0084	-	1	-	-
CYP4F11	0.0071	-	1	-	-
GCGR	0.0059	-	-	1	-
PHF15	0.0032	1	-	-	-
GCKR	0.0094	-	-	1	-
CRY2	0.0091	1	-	-	-
ZNF14	5.40E-09	-	-	-	-
ZNF24	0.0042	1	-	-	-
TLR3	0.0059	1	-	-	-
CUTL2	0.00013	-	1	1	-
ACTR3	0.0049	1	1	-	-
PCYT2	-	-	1	1	-
MMD	8.10E-05	-	1	-	-
ELL2	-	1	-	-	1
LGALS8	0.0086	1	-	-	-
CTBS	0.0024	-	-	1	-
HAGH	0.0015	-	1	-	-
CCDC98	1.80E-06	-	-	-	-
HCG_1983332	1.10E-07	-	-	-	-
DPYS	2.10E-05	-	1	-	-
EML4	8.20E-07	-	-	-	-
C4BPB	0.0021	-	1	1	-
MTSS1	-	1	1	-	-
NTS	-	-	1	1	-
RBM24	1.30E-07	-	-	-	-
RBM34	0.0048	-	-	1	-
DNAJC7	0.0054	1	-	-	-
CCDC6	8.80E-06	-	-	1	-
RIOK1	7.00E-08	-	-	-	-
OTC	0.00012	-	1	-	-
HMGA1	7.00E-09	-	-	-	-
HMGB2	0.0075	-	1	-	-
HMGCR	-	1	-	1	-
RPS18	0.00011	-	1	-	-
TPBG	1.10E-07	-	-	-	-
RPS3A	0.00069	-	1	-	-
WDR23	0.00074	-	-	1	-
PLG	0.00051	-	-	1	-
SMARCA5	0.0068	1	-	-	-
PBX1	-	1	1	-	-
PCCB	0.0011	-	1	-	-
PCK1	-	-	1	1	-
SELENBP1	0.01	-	1	1	-
PBEF1	0.00075	1	-	-	-
C1ORF9	0.0017	1	-	-	-
MPDZ	0.0096	-	1	-	-
GJB1	-	-	1	1	-

CYP4V2	2.00E-06	-	-	-	-
ADD3	0.0064	-	-	1	-
SET	0.002	-	1	-	-
PES1	0.0018	1	-	-	-
NDRG2	0.00042	-	1	-	-
LOXL2	0.0016	-	-	1	-
SMURF2	0.0057	-	-	-	1
NFKB2	0.0086	-	-	1	-
PGM2L1	0.0011	1	-	-	-
GLYAT	5.80E-06	1	1	-	-
SUCLG1	1.70E-05	-	-	1	-
TTK	-	1	1	-	-
ABCC2	0.00044	-	1	-	-
DUSP6	0.0053	1	-	-	-
