# Supplementary material for: Clinicopathologic and gene expression parameters predict liver cancer prognosis
Source: BMC Cancer. 2011 Nov 9;11:481. doi: 10.1186/1471-2407-11-481 (PMC3240666; doi:10.1186/1471-2407-11-481)

Figure S5, Predicting HCC Prognosis using Clinicopathologic Parameters + Gene Expression Profiles of Normal or Tumor Tissue

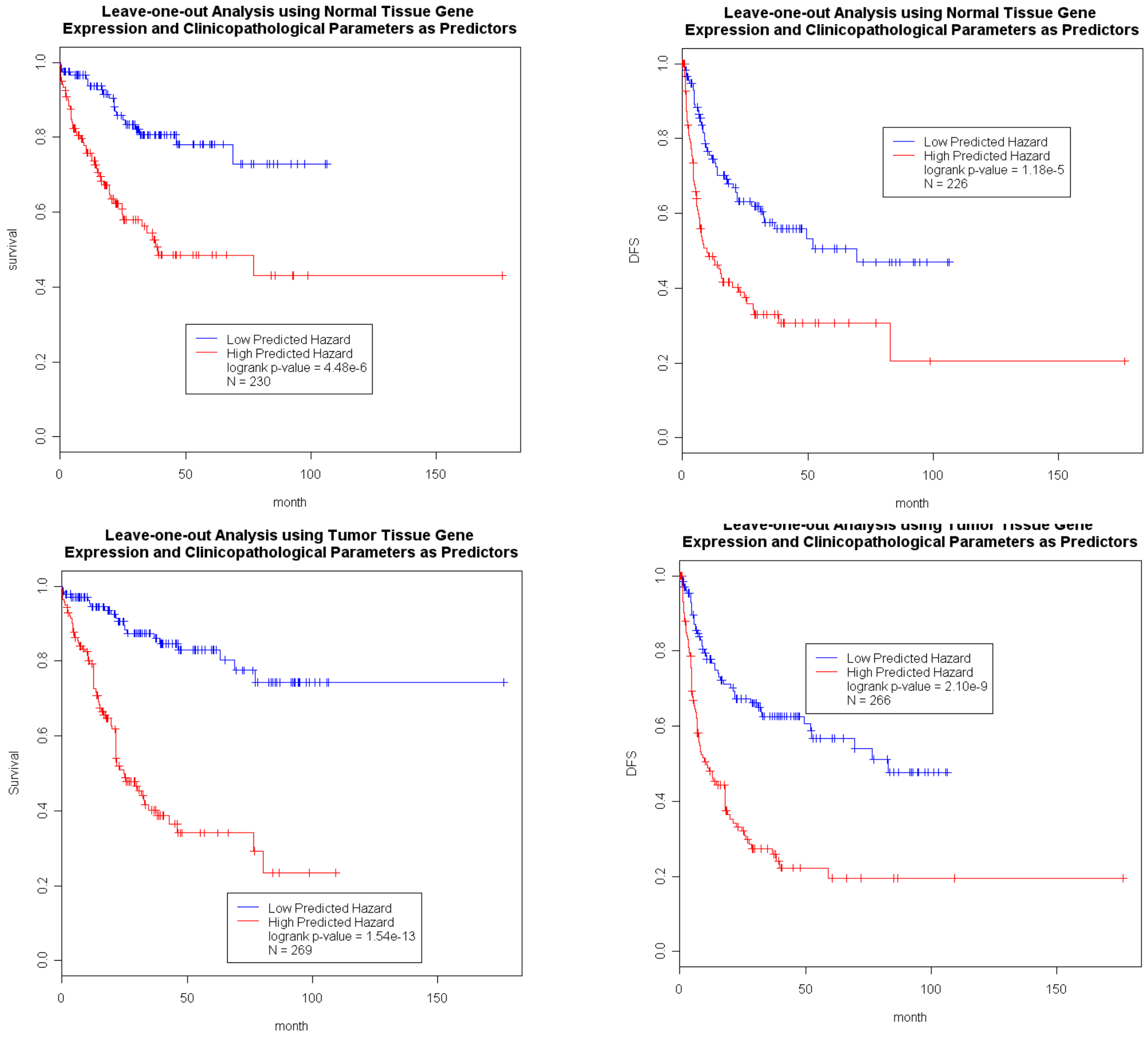

Supplement: Additional file 11 — Figure S5. Predicting HCC Prognosis using Clinicopathologic Parameters + Gene Expression Profiles of Normal or Tumor Tissue [file 1471-2407-11-481-S11.PDF]
