# Supplementary material for: Clinicopathologic and gene expression parameters predict liver cancer prognosis
Source: BMC Cancer. 2011 Nov 9;11:481. doi: 10.1186/1471-2407-11-481 (PMC3240666; doi:10.1186/1471-2407-11-481)

Figure S6, Predicting HCC Prognosis using Clinicopathologic Parameters + Gene Expression Profiles of Both Normal and Tumor Tissue

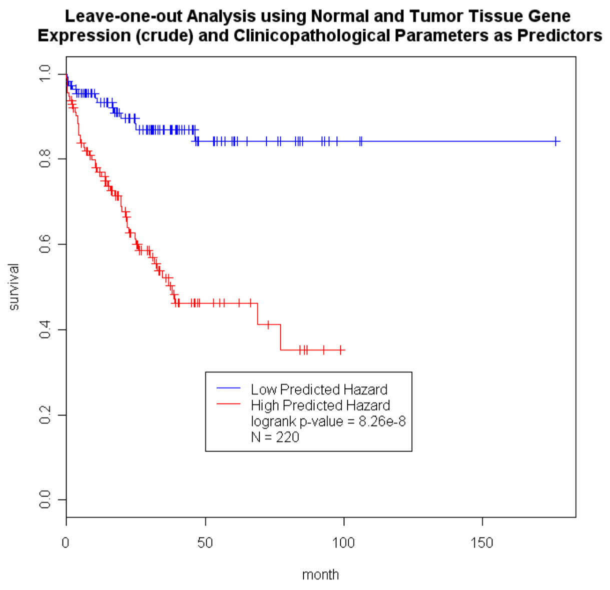

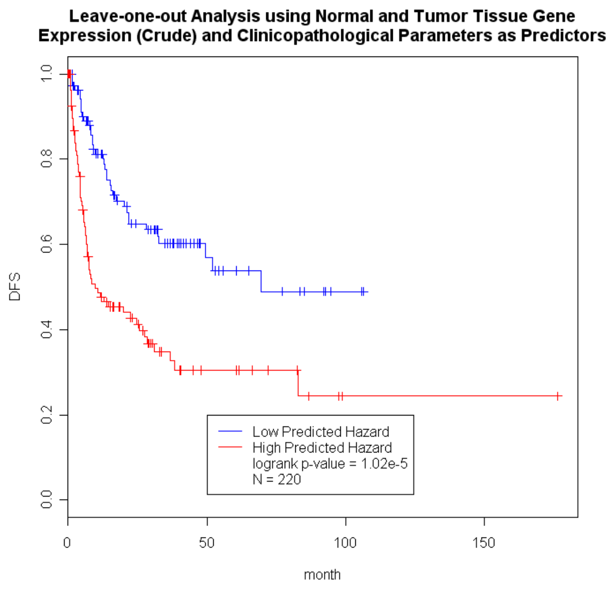

Supplement: Additional file 12 — Figure S6. Predicting HCC Prognosis using Clinicopathologic Parameters + Gene Expression Profiles of Both Normal and Tumor Tissue [file 1471-2407-11-481-S12.PDF]
